# Molecular mechanisms of chaperone‐directed protein folding: Insights from atomistic simulations

**DOI:** 10.1002/pro.4880

**Published:** 2024-02-26

**Authors:** Matteo Castelli, Andrea Magni, Giorgio Bonollo, Silvia Pavoni, Francesco Frigerio, A. Sofia F. Oliveira, Fabrizio Cinquini, Stefano A. Serapian, Giorgio Colombo

**Affiliations:** ^1^ Dipartimento di Chimica, Università di Pavia Pavia Italy; ^2^ Department of Physical Chemistry R&D Eni SpA San Donato Milanese Italy; ^3^ Centre for Computational Chemistry, School of Chemistry, University of Bristol Bristol UK; ^4^ Upstream & Technical Services – TECS/STES – Eni Spa San Donato Milanese Italy

**Keywords:** allostery, atomistic simulations, Hsp90, molecular dynamics, protein folding

## Abstract

Molecular chaperones, a family of proteins of which Hsp90 and Hsp70 are integral members, form an essential machinery to maintain healthy proteomes by controlling the folding and activation of a plethora of substrate client proteins. This is achieved through cycles in which Hsp90 and Hsp70, regulated by task‐specific co‐chaperones, process ATP and become part of a complex network that undergoes extensive compositional and conformational variations. Despite impressive advances in structural knowledge, the mechanisms that regulate the dynamics of functional assemblies, their response to nucleotides, and their relevance for client remodeling are still elusive. Here, we focus on the glucocorticoid receptor (GR):Hsp90:Hsp70:co‐chaperone Hop client‐loading and the GR:Hsp90:co‐chaperone p23 client‐maturation complexes, key assemblies in the folding cycle of glucocorticoid receptor (GR), a client strictly dependent upon Hsp90/Hsp70 for activity. Using a combination of molecular dynamics simulation approaches, we unveil with unprecedented detail the mechanisms that underpin function in these chaperone machineries. Specifically, we dissect the processes by which the nucleotide‐encoded message is relayed to the client and how the distinct partners of the assemblies cooperate to (pre)organize partially folded GR during Loading and Maturation. We show how different ligand states determine distinct dynamic profiles for the functional interfaces defining the interactions in the complexes and modulate their overall flexibility to facilitate progress along the chaperone cycle. Finally, we also show that the GR regions engaged by the chaperone machinery display peculiar energetic signatures in the folded state, which enhance the probability of partial unfolding fluctuations. From these results, we propose a model where a dynamic cross‐talk emerges between the chaperone dynamics states and remodeling of client‐interacting regions. This factor, coupled to the highly dynamic nature of the assemblies and the conformational heterogeneity of their interactions, provides the basis for regulating the functions of distinct assemblies during the chaperoning cycle.

## 1 INTRODUCTION

Protein folding and proteome maintenance in the crowded environments of cells are controlled by molecular chaperones, a class of molecular machines that interact with a plethora of substrate proteins, called clients, to guide them to their active forms. Chaperones Hsp90 and Hsp70, together with their associated role‐specific co‐chaperones, play key roles in these processes and oversee the correct folding and activation of clients, many of which are implicated in cell proliferation, survival, and adaptation pathways (Freilich et al., 2018; Hartl et al., 2011; Li & Buchner, 2013; Neckers & Trepel, 2014; Trepel et al., 2010). Chaperones of the heat shock proteins (Hsp) family undergo a complex conformational cycle to support client recognition, binding, folding, and release: the scheduling in the cycle is provided by nucleotide hydrolysis and the recruitment of co‐chaperones (Dahiya et al., 2022; English et al., 2017; Shiau et al., 2006; Smock & Gierasch, 2009; Zuehlke & Johnson, 2010). Dysregulation of these processes can lead to pathologies such as cancer or neurodegeneration (Schopf et al., 2017).

**FIGURE 1:**
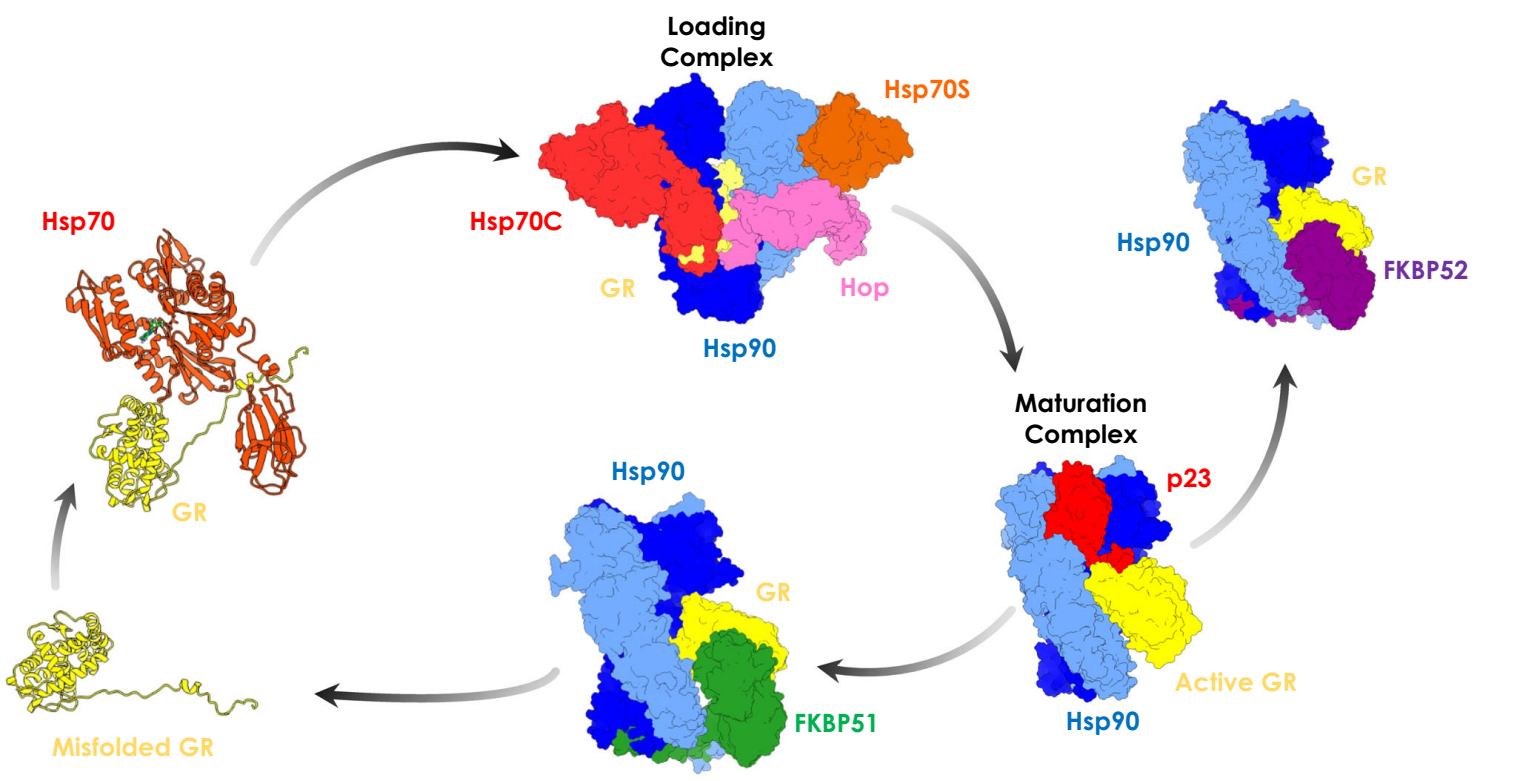
Overview of the glucocorticoid receptor (GR) chaperoning cycle. The unfolded GR is recruited by Hsp70, forming the first multiprotein complex. Then, GR and Hsp70 are loaded onto chaperone Hsp90, which, together with another Hsp70 molecule and co‐chaperone Hop, forms the Loading Complex. Subsequently, Hsp70s and Hop are released and co‐chaperone p23 binds. GR is remodeled from its inactive form to the active one in this step. After activation, there is another co‐chaperone switch, where p23 is released, and FKBP51 or FKBP52 are recruited (not studied here).

A first simplified and schematic overview of chaperone‐directed client folding depicts Hsp70 as an early‐acting chaperone that binds misfolded proteins, while Hsp90 acts further down the line on partially folded clients (Genest et al., 2019). Early events in the Hsp70‐Hsp90 chaperoning cycle comprise the binding of co‐chaperone Hop (Hsp Organizing Protein; Figure 1, in pink) to the open conformation of Hsp90 (Southworth & Agard, 2011). Hop, in turn, assists client transfer from Hsp70 to Hsp90 by binding each chaperone with unique TPR domains (Bhattacharya & Picard, 2021; Schmid et al., 2012).

Biochemical and biophysical investigations have revealed several mechanistic details on chaperone‐mediated folding cycles, which entail the identification of interactors, the timing of distinct enzymatic activities, and the roles of additional regulatory factors (Sahasrabudhe et al., 2017). However, until a few years ago, any detailed structural understanding of how clients interact with Hsp70/Hsp90 and their co‐chaperones had been missing.

The advent of Cryo‐EM has had a transformative impact in the field. Structural work illuminated various multiprotein complexes comprising, for instance, Hsp90 and co‐chaperones or substrates (Martino et al., 2018; Muñoz‐Hernández et al., 2020; Oberoi et al., 2022). The Agard Lab solved the structures of assemblies that are populated at different steps of the maturation cycle of different clients, particularly of the glucocorticoid receptor (GR). They provided experimental structures of the Hsp90:Hsp70C_ADP_:Hsp70S_ADP_:Hop:GR (Loading Complex (Wang et al., 2022)) and of the Hsp90_ATP_:p23:GR_DEX_ (Maturation Complex (Noddings et al., 2022)), representing key steps toward GR activation.

GR mediates the action of glucocorticoids in the cell, regulating glucocorticoid‐mediated gene expression. Alterations in glucocorticoid levels are linked to pathological conditions, such as Cushing's and Addison's disease (Kadmiel & Cidlowski, 2013).

**FIGURE 2:**
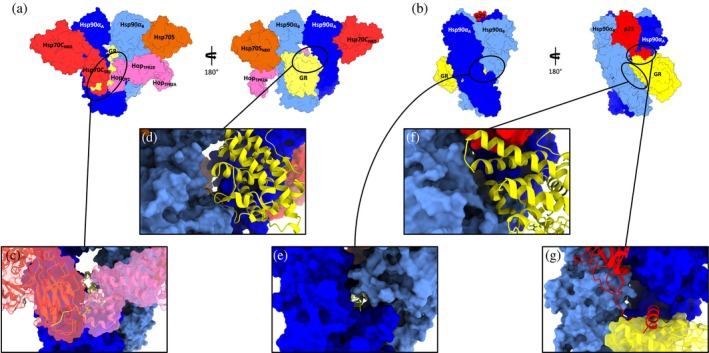
Detailed view of the loading and maturation structures with protein and domain subdivisions, highlighting the main discussed inter‐protein interfaces. (a) Front and rear view of the Loading Complex. Chaperone Hsp90 in blue (protomer A in blue and protomer B in light blue), Hsp70C in red, Hsp70S in orange, the co‐chaperone Hop in pink, and the client glucocorticoid receptor (GR) in yellow are shown. (b) Front and rear view of the maturation complex. Chaperone Hsp90 in blue (protomer A in blue and protomer B in light blue), co‐chaperone p23 in red and the client GR in yellow are shown. (c) A zoom‐in on the interface of the GR tail in the lumen of the Loading Complex. (d) Overview of the interface between the globular part of GR and chaperone Hsp90 in the Loading Complex, highlighting the GR portion that goes into the lumen. (e) Magnification on the interface of GR tail in the lumen of the maturation complex. (f) Overview of the interface between the globular part of GR and chaperone Hsp90 in the maturation complex, highlighting the GR portion that goes into the lumen. (g) Highlighting the interface between the p23 tail helix and the other components of the maturation complex, namely Hsp90 and GR.

Mechanistically, early‐stage GR chaperoning involves both Hsp70 and Hsp90, which, together with co‐chaperone Hop, assemble the so‐called “Loading Complex” (Hsp90:Hsp70C_ADP_:Hsp70S_ADP_:Hop:GR, PDB code: 7KW7; (Wang et al., 2022) Figure 1), where Hsp70C is dedicated to client‐loading and Hsp70S performs a scaffolding role. In this complex, both Hsp70s are in the high‐affinity ADP bound state. In this complex, Hsp90 adopts a peculiar intermediate “semi‐closed” conformation, where the N‐Terminal Domains (NTDs) are in an ATP‐bound‐like conformation, but not yet in the fully closed ATPase‐competent state (Figure 1, protomer A in blue protomer B in cyan). This configuration is stabilized through interfaces formed by Hsp90_NTD/M‐Domain_ and both Hsp70_NBD_s (Figure 1, Hsp70C in red, Hsp70S in orange). GR (only resolved in its *C*‐terminal ligand‐binding domain; Figure 1, in yellow) is still partially unfolded, with the folded region on one side of Hsp90 (Figure 2a, zoom in panel d) while the unstructured *N*‐terminal portion (toward the unresolved DNA‐binding and modulatory domains) threads through the “clamp lumen” between two Hsp90 M‐Domains, in a way that is reminiscent of the Hsp90α:Cdc37:Cdk4 complex (PDB code: 5FWL) (Verba et al., 2016). The GR's *N*‐terminal sequence (residues 519–531; numbering from PDB: 7KW7) contacts Hop_DP2_ and Hsp70C_SBD_ while the subsequent GR_helix1_ (residues 532–539; numbering from PDB: 7KW7) interacts with Hop_DP2_ and both Hsp90_M‐Domain_s (Figure 2a, zoom in panel c).

GR maturation occurs upon Hsp70s and Hop release and progression to a second complex, called the “Maturation Complex,” which comprises Hsp90 and co‐chaperone p23. Here, GR is observed in a conformation closer to the biologically functional active state, notably with a steroid molecule in the binding site (dexamethasone, DEX) (PDB: 7KRJ; (Noddings et al., 2022) Figure 1). Interestingly, compared to the Loading Complex, GR in this case is characterized by a remodeled *N*‐terminal portion. The terminal α‐helix is pulled out of the lumen and is folded onto the ligand‐binding domain (Figure 2b, zoom in panel f), while the pre‐helix portion interacts through the lumen (Figure 2b, zoom in panel e). The folded domain interacts with p23 and is stabilized through direct interactions with the co‐chaperone's *C*‐terminal α‐helix (Figure 2b, zoom in panel g). The Hsp90‐p23 interface mainly entails Hsp90_NTD_s and it stabilizes the fully closed ATP‐competent state (Noddings et al., 2022).

The resolution of the GR Loading and maturation complexes provides a privileged view of the molecular structures that underpin chaperone‐mediated client remodeling mechanisms. This knowledge could be significantly enriched if it could be exploited to unveil the key (microscopic) functional dynamic states populated by the various components of the assemblies in the different steps of the maturation cycle thus shedding light on the mechanisms of dynamic coordination among the partners. Such mechanistic information, being at the intersection of the key aspects of the functioning of the different complexes, could reveal the structural and dynamic determinants of the organization of the assemblies and the regulation of the activities of the various proteins involved.

In this work, we combine multiple microsecond‐long all‐atom molecular dynamics (MD) simulations of the loading and maturation complexes with advanced methods of analysis (ranging from internal dynamics analysis to perturbative approaches).

Our aim is to shed light on the common and differential aspects of the motions of the proteins, and their functional substructures (e.g., nucleotide binding sites, protein–protein interactions, and client binding sites) in the distinct complexes, how they are regulated by interactions with other partners, and the role of endogenous ligand binding (e.g., ATP and ADP). In this context, we also ask whether, how, and to what extent the dynamics of the regions of GR involved in the folding process are specifically modulated by the interplay with the folding machinery. We complement analyses of the wild‐type complexes with simulations where we investigate modifications in Hop that are known to compromise client processing. Next, we ask whether recognition in the Loading and Maturation Complexes depends on a general unfolding of the substrate or whether it depends on the tendency of specific client regions to undergo unfolding fluctuations. Finally, we exploit the data on internal dynamics and long‐range communication patterns in the large complexes at different stages of nucleotide processing to identify potential ligand binding pockets: the goal is to provide a structural basis of the design of chemical tools able to allosterically modulate the foldase activities of the complexes in a selective fashion. To this end, we focus on areas of the protein complexes that show direct communication with the client.

Our results are then used to build a model that sheds light on how distinct ligand‐states determine differential dynamic profiles for the functional interfaces defining the interactions in the complexes and modulate their overall flexibility to facilitate progress along the chaperone cycle. Interestingly, we find that the GR regions engaged by the chaperone machinery coincide with the ones particularly prone to partial unfolding fluctuations in the native state.

## 2 RESULTS

Here, we focus on the two key multiprotein complexes regulating the maturation cycle of GR. The Loading Complex (Hsp90α:Hsp70C_ADP_:Hsp70S_ADP_:Hop:GR) was simulated in equilibrium conditions in two different states, namely with ADP bound to the Hsp70s (simulations labeled Loading‐ADP, see Table 1) and with both Hsp70s in the apo state (simulations labeled Loading‐APO, see Table 1). The Maturation Complex (Hsp90_ATP_:p23:GR_DEX_) was also simulated in the experimental ATP‐bound state in Hsp90 with dexamethasone (DEX) bound to GR (simulations labeled Maturation‐ATP, see Table 1).

**TABLE 1:** Summary of the studied systems. This table contains information about the simulated systems, the structures used as the starting point for the simulations, and the length and number of replicates performed in each case. The following data are reported: the protein system name, the label that is used to refer to a specific simulation, the PDB code of the starting structure and information on the number of replicas, the length of each simulation, and the total length of each combined metatrajectory.

Label	Protein system	PDB ID starting structure	Number of replicas	Simulation length	Total length
Equilibrium molecular dynamics					
Loading‐ADP	Hsp90:Hsp70C_ADP_:Hsp70S_ADP_:Hop:GR	7KW7	4	1 μs	4 μs
Loading‐APO	Hsp90:Hsp70C_APO_:Hsp70S_APO_:Hop:GR	7KW7	4	1 μs	4 μs
Loading‐Y354E	Hsp90:Hsp70C_APO_:Hsp70S_APO_:Hop_Y354E_:GR	7KW7	4	1 μs	4 μs
Loading‐pY354	Hsp90:Hsp70C_APO_:Hsp70S_APO_:Hop_pY354_:GR	7KW7	4	1 μs	4 μs
Maturation	Hsp90:p23:GR	7KRJ	4	1 μs	4 μs
GR‐loading	GR from loading complex	7KW7	4	1 μs	4 μs
GR‐maturation	GR from maturation complex	7KRJ	4	1 μs	4 μs
GR‐alone	GR stand‐alone	5NFP	4	1 μs	4 μs
Non‐equilibrium molecular dynamics					
D‐NEMD‐loading	Hsp90:Hsp70C_ADP_:Hsp70S_ADP_:Hop:GR (ADP to APO)	7KW7	176	20 ns	3.52 μs
D‐NEMD‐maturation	Hsp90:p23:GR (ATP to ADP)	7KRJ	176	20 ns	3.52 μs

The goal of these equilibrium simulations is to establish whether specific substructures of the interacting partners feature distinctive dynamic properties that can be linked to client recognition, stabilization, and remodeling activities, and whether a conformational response to the nucleotide state is directed toward functional interface regions, especially involving proteins that are different from the ones the nucleotide is bound initially to.

For this, we started by characterizing the degree of internal coordination among all residue pairs in each assembly in different nucleotide states. Coordination is recapitulated by the profiles of the fluctuations in the distances (Distance Fluctuation (DF) parameter; cf. *Methods* [Section 5]) of all amino acid pairs in the complexes. This analysis sheds light on the collective internal dynamic effects linked to modifications in ligand states or residue mutation/post‐translational modification, and the mechanisms of dynamic structural adaptation of the various binding partners (Moroni et al., 2018; Morra et al., 2012; Rinaldi et al., 2018). Importantly, it reports on both local and long‐distance coordination mechanisms, the latter expected to be important in such large and variegated complexes. The DF parameter can consequently provide information about the dynamic redistributions of interactions in the assemblies in different conditions. The (ensembles) of residues that show differential profiles in the different states represent potential responsive hot spots sensing the ligand state. On the other hand, the (ensembles) of residues where the same dynamic profiles are consistently present can expectedly correspond to preorganized substructures that are important for regulating partner recognition. In this context, differences in the internal profiles for the same protein in different complexes and/or ligand states can highlight the protein's capacity to explore alternative dynamic states and unveil the mechanisms of plastic adaptation to different biochemical requirements.

To complement these data, we next used dynamical non‐equilibrium (D‐NEMD) simulations to model the consequences of ADP unbinding in the Loading Complex (Loading‐ADP) and ATP hydrolysis in the maturation complex (Maturation‐ATP) (see simulation labels in Table 1). This approach, combining MD simulations in equilibrium and non‐equilibrium conditions, allows to determine the time‐dependent structural response of a system subjected to an external structural perturbation, the latter being the removal or the modification of the chemical state of the nucleotide.

Lastly, we focused on comparing the different conformational and dynamic states that GR populates in the two steps of the folding cycle modeled here, the tendency of specific regions in the native structure of GR to undergo unfolding fluctuations, and the impact of Hop mutations and post‐translational modifications (PTMs) on the functional dynamics of the Loading Complex.

### 2.1 The dynamics of the loading complex in different nucleotide states

**FIGURE 3:**
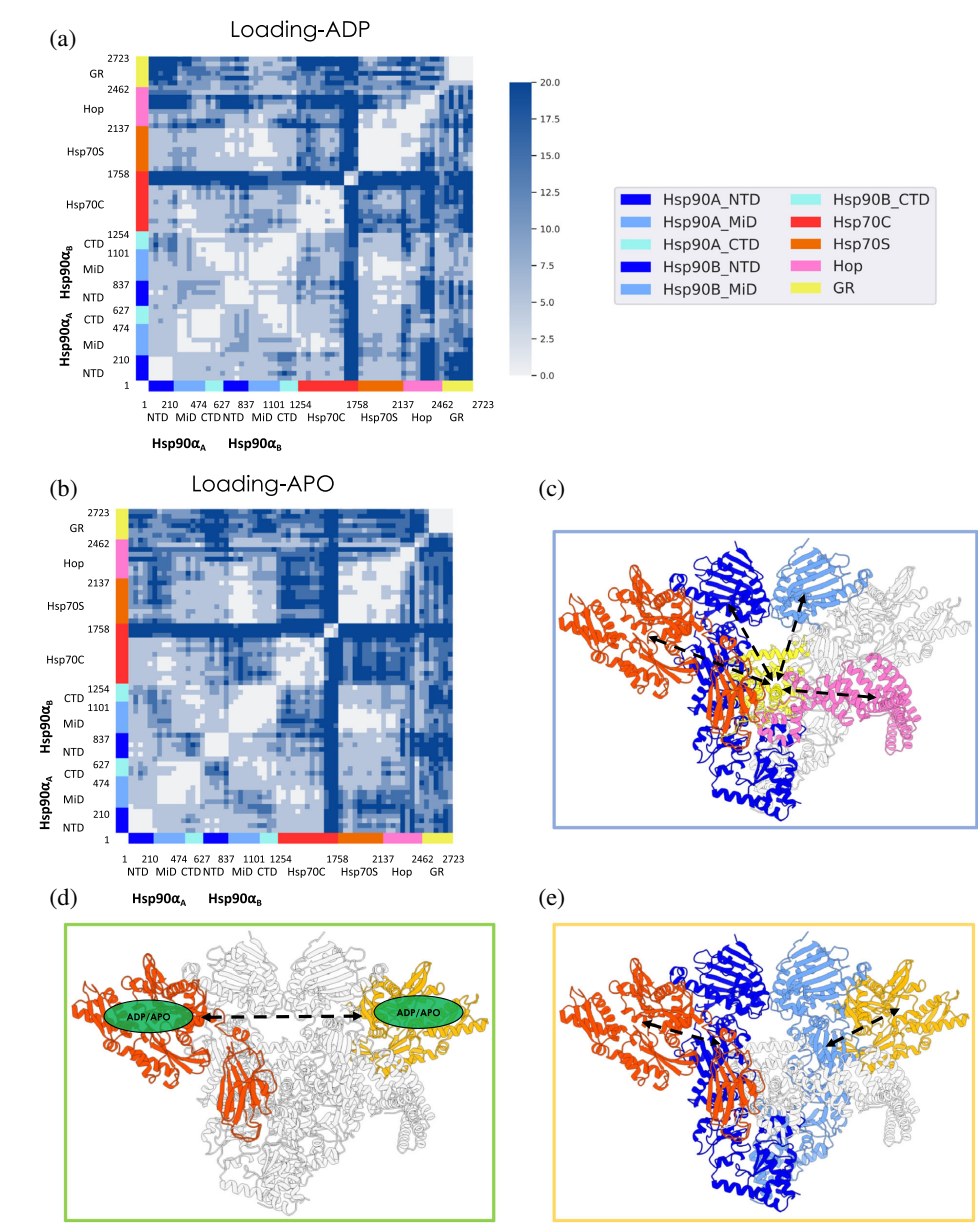
Simplified residue‐pair distance fluctuations matrix of the (a) Loading‐ADP system and of the (b) Loading‐APO system. DF values are averaged over 40 consecutive residue‐sequences (see *Methods* for details [Section 5]). Lighter pixels correspond to highly coordinated regions, while darker ones report on low coordination regions. To provide a structural projection of the differences in the internal dynamics of the two simulated systems, we report the major differences observed here and in the difference matrix (see Figure S1C) in subpanels (c), (d) and (e). In this view, colored areas represent the substructures and domains that display different coordination propensities between the Loading‐ADP and Loading‐APO system.

We begin by focusing on Loading‐ADP simulations. The fully detailed DF matrices are shown in Figure S1(A–C), while a simplified coarse‐grained version aimed at highlighting coordination patterns among domains and substructures in the complex (*see Methods* [Section 5]) is reported in Figure 3a,b. Moreover, we electronically provide a movie (https://drive.google.com/drive/folders/1-sKIgqZGgCPj7an04XuSxy-S8_Z6JDTu?usp=sharing) that shows the average coordination of each residue (calculated over the respective entire trajectory, see *Methods* [Section 5]) with every other residue in the complex. This provides a 3‐D dynamic representation of the DF matrix and internal coordination patterns in the various complexes (Morra et al., 2012). Interestingly, Hsp90 in this system is characterized by asymmetry in the dynamics of the two protomers. Internally, Hsp90's protomer B (henceforth Hsp90_B_) is highly coordinated with respect to protomer A (Hsp90_A_), whose N‐terminal Domain (NTD) is poorly coordinated with its M‐Domain and C‐terminal Domain (CTD) (cf. darker blue areas in Figure 3a, denoting higher DF scores). Interprotomer coordination between Hsp90_A_ and Hsp90_B_ is defined by patterns of low fluctuation between the NDBs of the two Hsp90 protomers, namely Hsp90_A:NTD_ and Hsp90_B:NTD_. Significant coordination is observed between Hsp90_A:small‐M‐domain_/Hsp90_A:CTD_ and Hsp90_B:small‐M‐Domain_/Hsp90_B:CTD_. On the other hand, low inter‐protomer coordination is detected between Hsp90_A:M‐Domain_/Hsp90_A:CTD_ and Hsp90_B:NTD_ as well as between Hsp90_A:NTD_ and Hsp90_B:M‐Domain_/Hsp90_B:CTD_.

The Hsp90 dimerized state and its internal dynamics are peculiar to the Loading Complex, where the NTDs are in a semi‐open arrangement and not correctly rotated to accommodate ATP. The fact that in this conformation of Hsp90, coupled dynamics is only observed between the two NTDs supports a model where the binding of a nucleotide at one protomer can be efficiently transferred to the other protomer, potentially poising the chaperone for binding the second nucleotide, necessary to trigger the subsequent closure of the clamp around the client. The absence of coordination between the NTDs and the client binding site in the M‐Domains (see Figure 3a) further suggests that the role of Hsp90 in the Loading Complex is primarily a scaffolding one, preorganized and ready to transition to an active foldase role.

Hsp70C_NBD_ is highly coordinated with Hsp90 compared to the other components of the complex. Interestingly, high coordination is more pronounced with Hsp90_A_, the directly bound protomer, compared to Hsp90_B_. Moreover, this analysis clearly highlights coordination between the active site area of Hsp70C and the active site region of Hsp90_A_. Hsp70C_NBD_ shows low coordination with Hsp70S (except for mutual communication between domains Hsp70S_NBD–IIA/IIB_ and Hsp70C_NBD–IIA/IIB_), Hop, and the entire GR. Hsp70C_SBD_ shows a low‐coordination pattern with all other components of the complex except for Hop_DP2_ and the terminal region of the GR unfolded tail (from residue 521 to residue 529; numbering from PDB: 7KW7).

Similar to Hsp70C, Hsp70S (only Hsp70S_NBD_ is resolved) is highly coordinated with Hsp90, specifically with the directly bound protomer Hsp90_B_. Interestingly, residues surrounding the Hsp70S active site are highly coordinated with the Hsp90_B_ active site region. Hsp70S_NBD_ shows poor long‐range connection with Hsp70C_SBD_, while with Hsp70C_NBD_ it shows lower DF values. The coordination pattern for Hop and GR is more complex. In particular, Hop_TPR2A_ and Hop_DP2_ are rigidly coordinated with Hsp70S_NBD_, while Hop_TPR2B_ shows poor coordination. Focusing on the client, the unfolded tail is poorly coordinated with Hsp70S, while the folded GR's domain shows generally higher coordination with Hsp70S_NBD_ compared to Hsp70C_NBD_. Interestingly, the observed connection between Hsp70C_NBD_ and Hsp70C_SBD,_ in combination with the coordination between Hsp70C_SBD_ and GR, suggest that, in this complex, Hsp70C's role is to stabilize and pre‐organize GR for further steps: the unfolded part of the substrate is complexed by Hsp70C_SBD_, which in turn directly communicates with the nucleotide‐bound domain. This organization could facilitate an efficient transmission to the substrate protein of nucleotide modification/exchange events in the NBD. Hsp70S appears to be dedicated to mediating the communication between Hsp90 and Hop.

Hop_TPR2A_ is highly coordinated with Hsp90_B:NTD_/Hsp90_B:M‐Domain_ and with Hsp70S. Hop_TPR2B_ shows a general low‐coordinated pattern with all the components, compared to Hop_TPR2A_. Hop_DP2_ is highly correlated with Hsp90_A:M‐domain_/Hsp90_A:CTD_ and Hsp90_B:M‐Domain_/Hsp90_B:CTD_, while with the NTD of both protomers DF values are higher. Notably, Hop_DP2_ and GR unfolded tail are rigidly interconnected (despite the GR region passing through the Hsp70C_SBD_).

Focusing on the dynamics of the client protein, GR's unfolded tail is rigidly coordinated with Hsp70C_SBD_ and with Hsp90_M‐Domain_/Hsp90_CTD_ of both protomers (particularly with protomer B). In particular, GR residues 521 to 529 of GR are highly connected with Hsp70C_SBD_, while GR residues 530 to 551 are with Hsp90_M‐Domain_/Hsp90_CTD_ (residues numbering from PDB: 7KW7). This coupling between the unfolded region of the client and the functional domains of the chaperones may protect and sequester the partially unfolded client while simultaneously prompting it for successive mechanical remodeling. It is possible to observe rigid coordination between the GR's folded domain and Hsp90_B:M‐Domain_/Hsp90_B:CTD_, which act as scaffolding domains. Interestingly, the GR folded domain is almost constantly coordinated with the whole Hsp70C.

Turning to the Loading‐APO system, where both ADP molecules were removed from the Hsp70s prior to the simulations, it is interesting to observe that the removal of the nucleotide induces a general decrease in internal coordination compared to the previous case (Figure 3b and Figure S1(B,C)). In Hsp90, the two protomers show a differential response to ADP removal: Hsp90_A:NTD_ becomes less strongly coordinated to Hsp90_A:M‐Domain_ and Hsp90_A:CTD_, while Hsp90_B:NTD_ gains coordination with Hsp90_B:M‐Domain_ and Hsp90_B:CTD_. Moreover, Hsp90_A:M‐Domain_ and Hsp90_B:M‐Domain_ lose mutual coordination (M‐Domain is where client binding occurs) (see Movie provided electronically; https://drive.google.com/drive/folders/1-sKIgqZGgCPj7an04XuSxy-S8_Z6JDTu?usp=sharing).

Overall, the major differences in internal dynamics concern Hsp70C, Hsp70S, Hop, and GR. Hsp70C_NBD_ and Hsp70C_SBD_ show markedly less cooperative dynamics. Upon ADP removal, Hsp70C_NBD_ becomes more coordinated to Hsp90_B:M‐Domain_/Hsp90_B:CTD_, Hsp70S (except for residues from 75 to 115; numbering from PDB: 7KW7 ^20^), and Hop_TPR2A_. The communication between the Hsp70 molecules, even if distal in the Loading Complex, appears thus to be highly dependent on the nucleotide states (Figure 3b and Figure S1(B,C); see Figure 3d for a schematic view). On the other hand, Hsp70C_NBD_ is highly coordinated with Hop_TPR2B_ and GR. Hsp70C_SBD_ coordination becomes low with Hsp90_A_, Hsp90_B_, Hop_DP2,_ and of particular interest with GR. These results underline the importance of nucleotide (un)binding in regulating the long‐distance allosteric cross‐talk between the chaperones and clients.

Upon ADP removal, Hsp70S becomes low coordinated to Hsp90_A:NTD_/Hsp90_A:M‐Domain_ and with Hsp70C_NBD_. Interestingly, Hsp70S loses the high coordination pattern with GR, as opposed to Hsp70C.

Notably, the cross‐talk between Hop_TPR2A_ and Hop_TPR2B_, which was shown to be crucial to Hop's function (Bhattacharya et al., 2020; Bhattacharya & Picard, 2021; Castelli et al., 2023), is substantially silenced in Loading‐APO. Moreover, coordination with GR becomes low in this system, with the other components of the complex, namely Hop_TPR2A_ and Hop_TPR2B_ showing an opposite behavior. TPR2A loses the high coordination pattern with Hsp90_B_ and Hsp70C, while TPR2B gains coordination with Hsp90_B_, Hsp70C, and Hsp90_A_. Hop_DP2_ loses coordination with Hsp90_A_, Hsp90_B,_ and Hsp70C_SBD_.

Of note, GR loses coordination with the entire Hsp90_B_, Hsp70C_SBD_, Hsp70S, and Hop (Figure 3b Figure S1(B,C); see Figure 3 (C) for a schematic representation).

To summarize, we observe that different nucleotide states determine distinct patterns of internal dynamics. Comparing Loading‐ADP with Loading‐APO, the allosteric and long‐distance cross‐talk among the partners is modified all over and not only in the vicinity of the active sites of Hsp70 (e.g., between Hsp70s and Hsp90 client binding site; Figure 3a,b and Figure S1(C); see Figure 3e for a schematic representation). Interestingly, GR, while remaining connected to the various partners of the complex, fine‐tunes its dynamic behavior in the ADP versus APO states. Overall, this organization results in a delicate balance among the dynamic entities of the assembly, which appears to be dependent on the nucleotide state, forming a highly responsive machinery that converges on preparing the GR for the next folding steps.

### 2.2 Identification of nucleotide‐induced conformational signals

Next, we use D‐NEMD to identify and characterize the pathways associated with signal transduction from the active sites of Hsp70s. To this end, we performed 176 short non‐equilibrium simulations (*D‐NEMD*; for a total of 3.52 μs) starting from multiple conformations selected from the equilibrated part of the long simulations of the Loading‐ADP system (see *Methods* [Section 5] section for details). In each short non‐equilibrium simulation, ADP was removed from Hsp70's active sites, and the simulation was run for 20 ns. To extract the structural response of the proteins to the removal of ADP and identify the regions implicated in the transmission of these structural changes, we used the Kubo‐Onsager relation introduced by Ciccotti et al. (Ciccotti & Jacucci, 1975) (see the *Methods* section for details [Section 5]).

Of note, the sudden (artificial) removal of ADP is intended to “force” the system out of equilibrium and the emergence of the structural responses of the proteins as they adapt to empty Hsp70's active sites. Given the artificial nature of the perturbation (in this case, the instantaneous removal of the ADP molecules), the timescales of the observed response do not directly correlate with the biological timescale. Instead, the responses allow for the identification of the initial steps associated with signal transduction and reveal the regions of the assembly components that are most responsive to the perturbation. On longer timescales, these effects can combine and ultimately lead to the onset of large conformational events associated with nucleotide removal.

**FIGURE 4:**
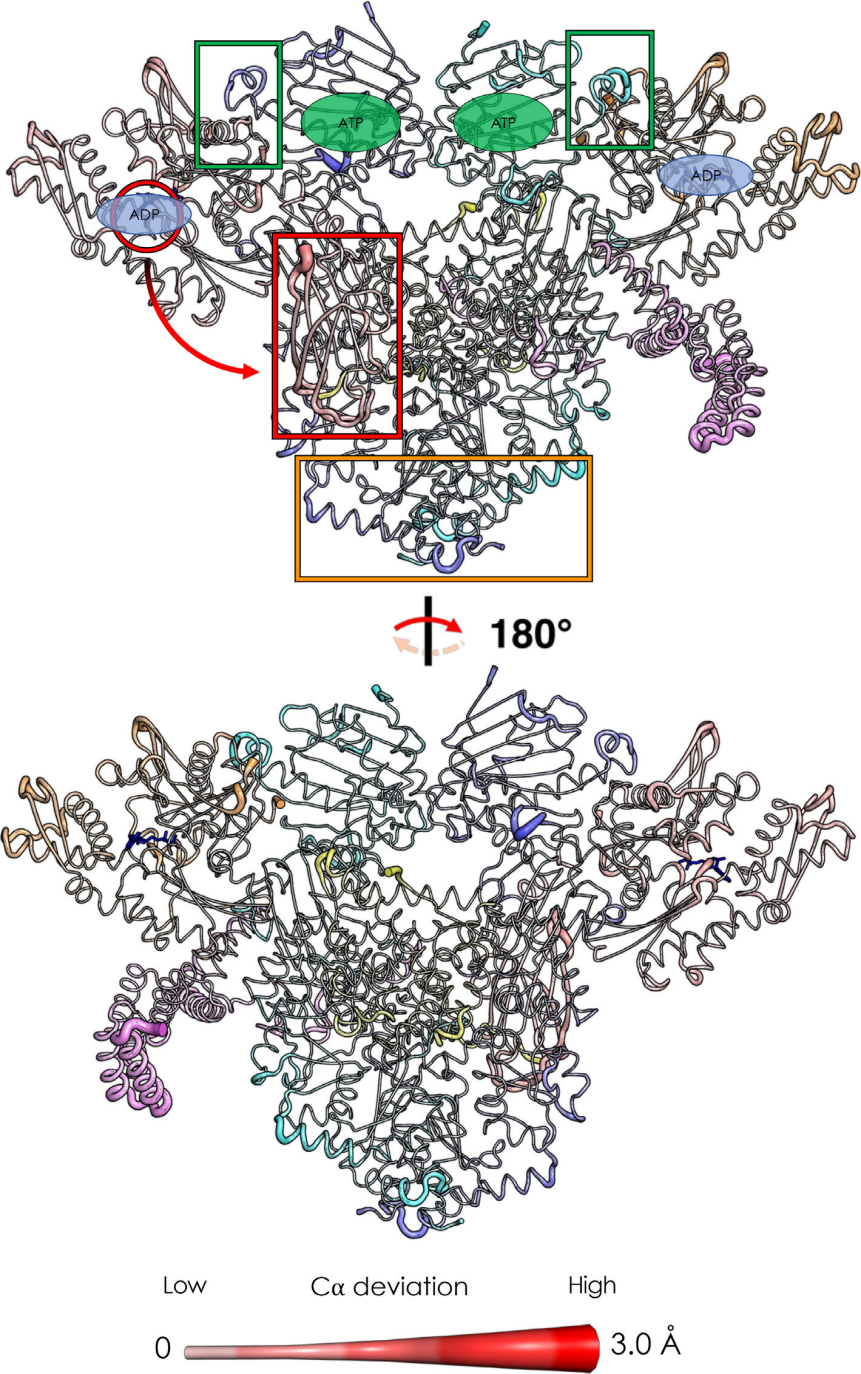
Structural and dynamic response induced by ADP removal in the Loading‐ADP structure. The orientations and atom color codes are the same as those for Figure 2a. Backbone thickness of each residue and color intensity is proportional to the average deviation of each C⍺ atom in the non‐equilibrium simulations—that is, after ADP removal—and its position in the equilibrium MD simulations at *t* = 5 ns. The green rectangles highlight the interfaces between the Hsp70_NBD_ and the Hsp90_NTD_ (adjacent to the nucleotide active site region). The orange rectangle highlights the signal propagation toward the CTD portion of the chaperone, showing the effect on the hinge region of the protein. The red rectangle and the arrow shows the signal reverberation to the Hsp70S_SBD_, where GR's unfolded tail binds the chaperone.

As expected, 5 ns after ADP removal, the active sites of both Hsp70S and Hsp70C are characterized by increased values of C‐alpha deviations, representing the onset of allosteric signaling (Figure 4, see also Figure S2). Interestingly, Hsp70C_SBD_ is one of the most affected regions of the complex, showing the relevance of Hsp70_NBD_/Hsp70_SBD_ allosteric connection and its tight nucleotide dependence, (Clerico et al., 2019; Swain et al., 2007) even in the context of a large biological assembly. In passing, we observe that the highest *RMSF* values (see Figure S3), calculated on the equilibrium trajectories, characterize ordered portions of secondary structure of Hsp70C, in particular its SBD. Here, the ADP‐bound state shows slightly lower values (16–18 Å) compared to the APO state (18–20 Å). Visual inspection of the trajectory clarifies the origin of such high values: we observe a flipping movement of the entire Hsp70C_SBD_ (where client binds) (1‐D RMSF data are color‐plotted on 3‐D structure in Figure S3; for a graphical representation of the dynamics see Figure S4). Importantly, we observed that ADP unbinding reverberates also on GR's unfolded tail. This long‐range effect can be explained through the direct interaction between the perturbed client portion and the Hsp70_SBD_, one of the regions most affected by ADP unbinding (Figure 4). Notably, Hsp90's loops at Hsp90_NTD_/Hsp70_NBD_ interfaces of both protomers (residues 60–75; numbering from PDB: 7KW7) are influenced by ADP removal. Of note, Hsp90_NTD_ loops are adjacent to the ATP binding site, supporting a direct coupling between the active sites of the two chaperones and nucleotide processing (Kirschke et al., 2014). In this context, mutations at the Hsp90_NTD_/Hsp70_NBD_ interface have been observed to impair client (GR, v‐Src, and luciferase) maturation (Bohen & Yamamoto, 1993; Doyle et al., 2019; Genest et al., 2015; Kravats et al., 2018; Nathan & Lindquist, 1995). Additional distant regions of the complex show correlations with ADP removal as the loops of the Hsp90 *C*‐terminal domain of both protomers (residues 552–570 and 648–657). During the next few nanoseconds, we observed an accumulation in the Cα deviations on all aforementioned protein regions.

These results combined with experimental evidence (Wang et al., 2022) support a mechanism whereby nucleotide‐regulated Hsp70 motions influence the progress of the client folding mechanism as well as the interplay between Hsp70 and Hsp90 nucleotide cycles. Importantly, the interactions at the Hsp90_NTD_/Hsp70_NBD_ have been experimentally observed to play a key role in defining the orientation of the Hsp90_NTD_ with respect to the Hsp90_M‐Domain_ (Genest et al., 2015; Wang et al., 2022). Our results show how these interactions and the motions of the involved substructures are remodeled by the presence/absence of the ligand in the Hsp70 binding site, exposing a possible mechanism that combines the conformational dynamics of the two chaperones.

### 2.3 Impact of mutations and post‐translational modifications in co‐chaperone Hop and their functional implications

We further tested the stability of the Loading Complex internal dynamics by introducing phosphorylation and single‐point mutations in the co‐chaperone Hop. Hop was previously shown to be sensitive to phosphorylation and mutation, which impair client maturation (Bhattacharya et al., 2020; Bhattacharya & Picard, 2021; Castelli et al., 2023). We then performed an additional set of equilibrium MD simulations of the Loading–ADP system comprising the following Hop variants: Hop‐pY354 and Hop‐Y354E (see Table 1).

In the Loading‐pY354 system, we observed a generalized rigidification of all the complex components (see Figure S5). However, some exceptions were identified and comprise functionally relevant regions. In particular, GR loses coordination with Hsp90_B:large‐M‐Domain_ and with Hsp70C_SBD_ (see Figure S5). Notably, these regions are both implicated in client binding and remodeling. Moreover, Hop_TPR2A_ and Hsp70S (residues 269–362; numeration from PDB: 7KW7) lose their cross‐talk with the client.

In the case of Loading‐Y354E, we observed a different effect on the internal dynamics (see Figure S6). The DF matrix shows a loss of coordination between the client GR and multiple complex components, in particular with various portions of Hsp90 forming the entire client binding site (Hsp90_A:CTD_, Hsp90_A:M‐Domain_,Hsp90_B:CTD_ and Hsp90_B:M‐Domain_), with the whole Hsp70S, with Hop_TPR2A_ and with Hsp70C_SBD_ (which take parts in client binding) (see Figure S6).

These results show once more how the complex interplay among the various partners is highly regulated and dependent on the specific states of the interacting proteins. Indeed, a single perturbation of key amino acids reverberates to functionally relevant regions of the multiprotein complex even at long distances.

### 2.4 Characterization and analysis of the maturation complex

**FIGURE 5:**
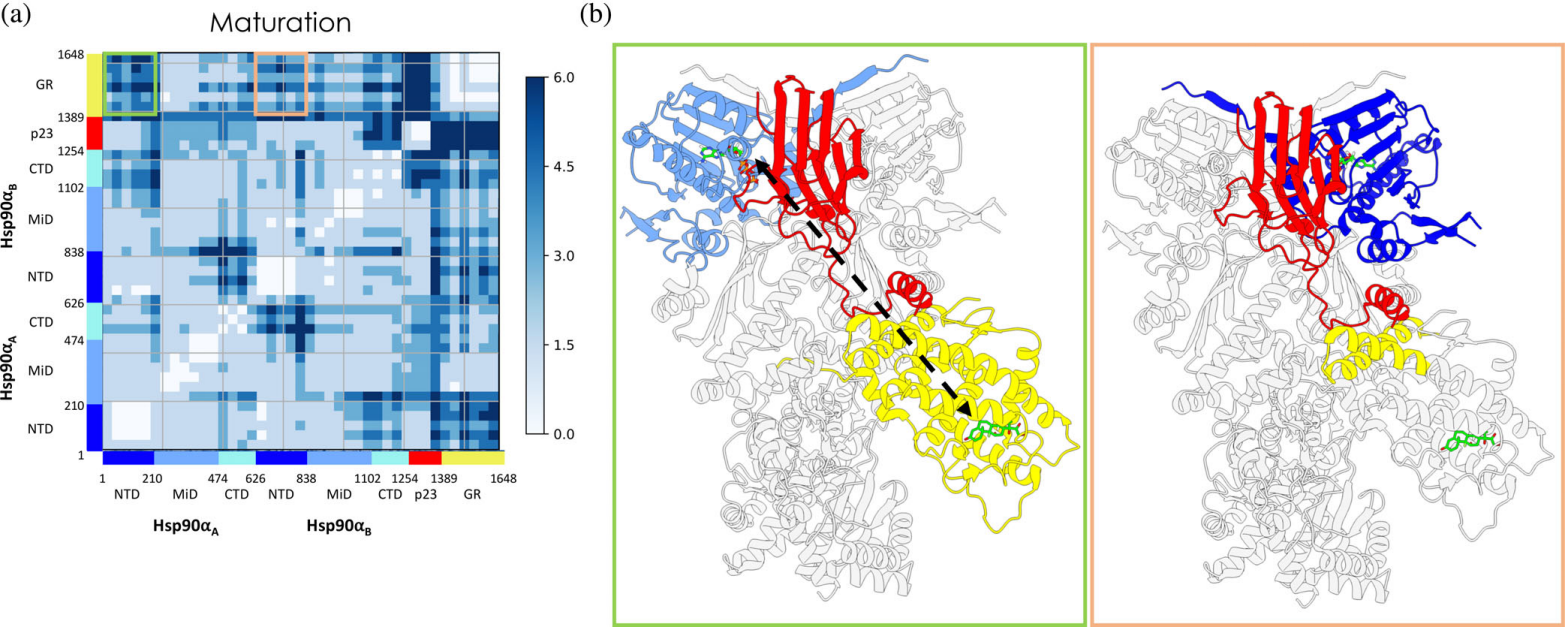
(a) Simplified residue pair distance fluctuations and (b) domain cross‐talk for the Maturation Complex in the ATP‐bound state. The axes report the residue numbers and domain organization. Lighter pixels correspond to highly coordinated pairs, while darker ones report on low coordination pairs. The colored rectangles represent the difference in coordination between the two protomers with the client GR. (b) A 3D schematic representation of the different coordination between NTD domains and GR. The green‐bordered scheme is referred to long‐range coordination for NTDB, while orange one refers to the patterns of NTDA. The NTD of protomer B displays stronger coordination with GR, that not only occurs at the interface with Hsp90 and p23 as in the case of protomer A but also extends the active site of the client.

Similarly to the Loading Complex, we also calculated the DF matrix for the Maturation‐ATP system. The full DF matrices are shown in Figure S7, while a substructure‐organized representation (*see Methods* [Section 5]) is reported in Figure 5a (for a schematic representation of key observations, see Figure 5b). We also electronically provide the DF movies (https://drive.google.com/drive/folders/1‐sKIgqZGgCPj7an04XuSxy‐S8_Z6JDTu?usp=sharing) showing the average coordination of each residue (calculated over the respective entire metatrajectory, *see Methods* [Section 5]) with every other residue in the complex. Overall, protomer B of Hsp90 is more strongly connected to p23 than protomer A. Protomer A, interestingly, is more coordinated to GR, underlining the importance of asymmetry in functional dynamics.

The block character of the matrices reflects the domain organization of the participant proteins. Interestingly, homologous domains from different Hsp90 protomers are characterized by distinct inter‐protein distance fluctuation patterns. Compared to Hsp90_A:NTD_, Hsp90_B:NTD_ is shown to be more connected to both p23 and GR. Moreover, Hsp90_A:M‐Ddomain_ has a higher number of residues whose dynamics is coupled to GR. The coordination with p23 is limited. Strikingly, Hsp90_B:M‐Domain_ behaves in the opposite way, that is, with prevalent coordination with p23 and low coordination with GR. Looking at the Hsp90_CTD_s, on the other hand, correlation with both p23 and GR remains low with respect to the previous two domains, although that of Hsp90_A:CTD_ is higher than that of Hsp90_B:CTD_. GR and p23 are never coordinated with each other, except for p23_tail helix,_ which shows coherent motions with the entire folded domain of GR. From a biological perspective, this evidence supports its role in pre‐organizing and scaffolding the client (Biebl et al., 2021). Overall, DFs reveal a marked difference in the behavior of protomer A or B in Hsp90 toward other components of the Maturation Complex.

To investigate the origins of this differential behavior in the two protomers, we carried out an in‐depth analysis of the areas around the active sites, as they are part of regions where the cycle‐regulating nucleotide processing takes place. Moreover, Cryo‐EM shows that GR is bound to a DEX molecule. Given this, we first specifically analyzed the DFs of residues located within a distance of 4 Å from these ligands, with all other residues in the complex. Both active sites of Hsp90 are characterized by low values of DFs, reflecting a high communication propensity, with the whole of p23. At the same time, there is evidence of a weak coordination between the region around the active site of Hsp90 protomer A (Hsp90_A_) and the portion of GR at the interface with Hsp90. In the case of the area close to the active site of protomer Hsp90_B_, areas of strong coordination with GR involve not only its interface region with Hsp90 but also extend to the active site of the client protein. Consistent with this view, residues around DEX in the binding site of GR show dynamic coupling with both protomers' M‐Domains and, more interestingly, with regions adjacent to the active site in the NTD of Hsp90_B_.

This highlights a distinction in the dynamic states of the two Hsp90 active sites, which could be indicative of a diversity of functional/regulatory roles for the two protomers. To corroborate this observation, the RMSDs of each Hsp90_NTD_ relative to each other were compared, first aligning the MD metatrajectory on the secondary structures of the whole complex except for one of the two NTDs, and then repeating the same procedure while excluding the other NTD. Plots of the distributions of the two RMSDs show clear differences (see Figure S8).

Visual inspection of the MD metatrajectory complements the information obtained above. The fact that the p23 tail helix contains residues that are among the most fluctuating ones and that, at the same time, this is the only portion of p23 to communicate with GR, supports a functional role for the motif (Biebl et al., 2021; Seraphim et al., 2015). Indeed, this helix, via its general flexibility, could mediate target recognition and recruitment during client loading on Hsp90. It could also play an active part in the folding stage by coordination with GR. Globally, co‐chaperone p23 directly acts on Hsp90 to modulate its ATPase activity to ensure proper client processing and sliding through the lumen. Indeed, p23 shows extensive coordination with the chaperone. Specifically, p23 shows low DFs with both Hsp90_NTDs_, where active sites reside, and with the Hsp90_B:M‐Domain_.

These results show a clear dual‐acting mechanism of the co‐chaperone p23, where its terminal alpha‐helix acts as a scaffold for GR, while the *C*‐terminal folded portion is rigidly connected to both Hsp90_NTD_s and promoting the fully closed state of the chaperone, allows longer time for client remodeling (Ali et al., 2006; Noddings et al., 2022; Prodromou & Bjorklund, 2022; Schopf et al., 2017). A further important aspect concerns the communication between the active site of Hsp90 and the client GR, where the chaperone may act in preserving GR activity.

### 2.5 The response of the maturation complex to ATP hydrolysis

**FIGURE 6:**
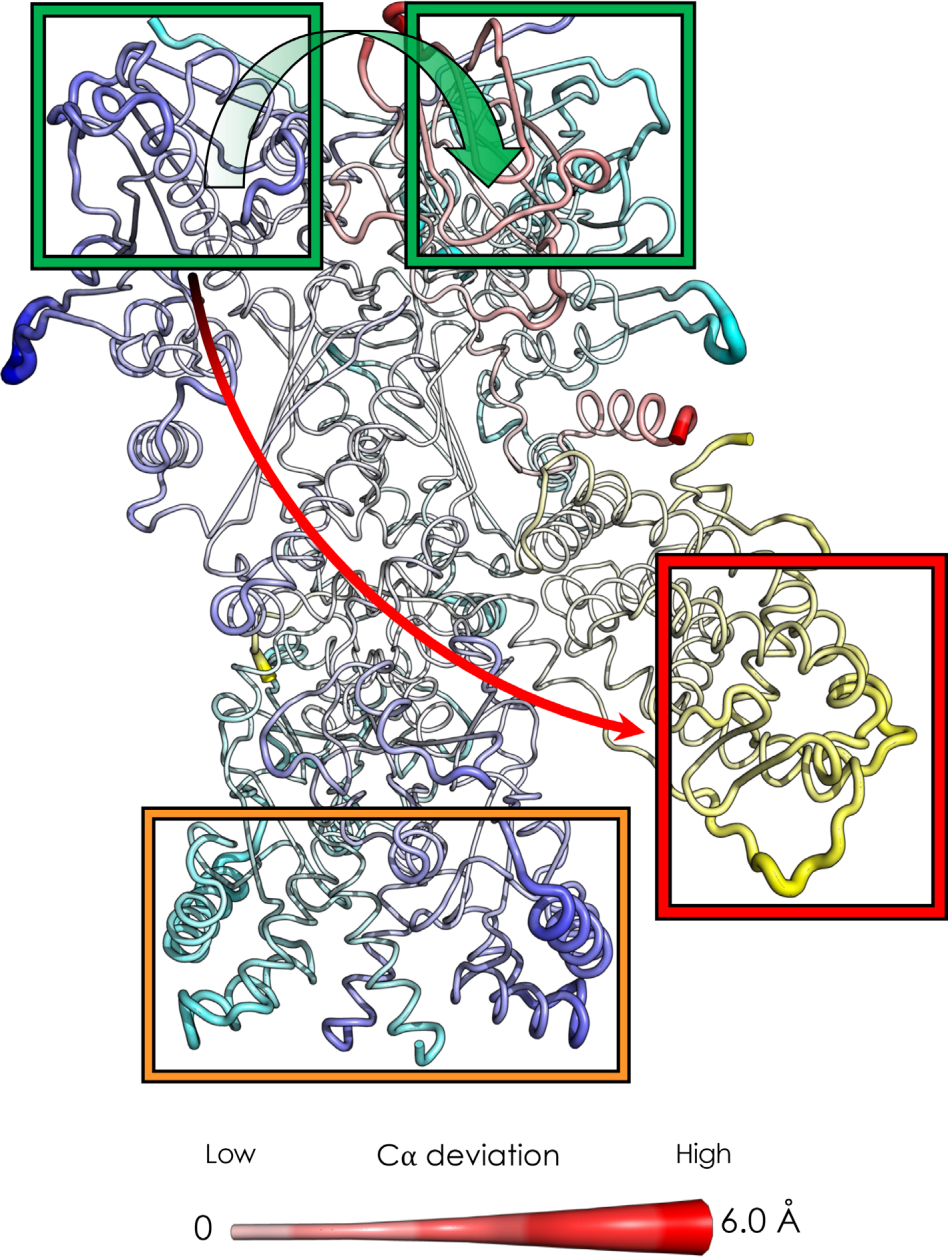
Structural and dynamic responses to ATP hydrolysis in the Maturation complex. The orientations and atom color codes are identical to those described in Figure 2b. Backbone thickness of each residue and color intensity is proportional to the average deviation of its C⍺ atom in D‐NEMD simulations—that is, after ATP hydrolysis—from its position in equilibrium MD simulations at *t* = 4 ns. The green rectangles highlight the two active sites of Hsp90. The green arrow shows the signal reverberation from protomer B active site to protomer A active site. The orange rectangle highlights the signal propagation toward the CTD portion of the chaperone, showing the effect on the hinge region of the protein. The red rectangle and the arrow shows the signal reverberation to the GR active site‐comprising region.

To decrypt the mechanisms of conformational signal transmission regulated by ATP binding and hydrolysis in the maturation complex, we performed D‐NEMD simulations, similar to the ones described above. Here, we have simulated the cleavage of the gamma‐phosphate from ATP to give ADP and inorganic phosphate as described in *Methods* (Section 5). We carried out 331 simulations of 20 ns each, corresponding to a total of 6.62 μs. Once again and similarly to what was described above, we used the Kubo Onsager relation (Ciccotti & Jacucci, 1975) to extract the response of the proteins and identify the regions implicated in signal transmission upon ATP hydrolysis in the active sites (see *Methods* section for details[Section 5]) 4 ns after perturbation (Figure 6 and Figure S9(A,B)). As expected, the Hsp90 NTDs are the most affected parts upon hydrolysis, specifically the active sites and the residues nearby. It is, however, much more interesting to observe that areas distal from the nucleotide sites emerge as responsive to the perturbation. Indeed, we note that ATP hydrolysis signaling accumulates in the *C*‐terminal domain of both protomers, where Hsp90 dimerization takes place, and the opening/closing motions of the clamp are hinged (Ratzke et al., 2010). Strikingly, we also observed that the signal is transmitted not only within a protein but also through protein–protein interfaces, such as Hsp90‐GR interface.

In this context, it is interesting to observe that one of the most affected regions is the area surrounding the dexamethasone molecules, namely the GR binding site. Finally, we observe that p23 is less involved in this signal transduction mechanism, as the effect of hydrolysis leads to fewer deviations. Notably, we observe an asymmetric behavior. If we compare the impact of nucleotide hydrolysis in the different sites, we can see that Hsp90_A_ has a more pronounced effect in the CTD region of both protomers with respect to Hsp90_B_. On the other hand, hydrolysis in Hsp90_B_ preferentially perturbs the previously cited regions of GR and p23 (see Figure S9(C)).

### 2.6 Characterization of GR dynamics in isolation: Implications for chaperone‐machinery engagement

To gain additional insights into GR dynamics, we simulated and compared the trajectories of GR structures isolated from the Loading Complex (GR‐loading), from the Maturation Complex (GR‐maturation), and alone in a native, fully folded conformation (GR‐alone) (see Table 1).

The folded portion of GR both in the GR‐Loading and the GR‐Maturation simulations shows great stability (RMSD around 1–2.5 Å; see Figure S10(A,B); red and blue lines), as shown by the time evolution of RMSD with respect to the native structure. The RMSD values for these runs are highly similar to those observed for the fully folded protein in the GR‐alone simulation (see Figure S10(C)). If we also consider the unfolded tail, high fluctuations are observed for the former two simulations, with RMSD values increasing (RMSD around 7–11 Å for the Loading Complex and 4–6 Å for the Maturation complex) (see Figure S10(A,B); black and green lines). Considering only the folded portion, a reduction in the RMSD values can be observed upon passing from the GR‐Loading to the GR‐Maturation and then to the GR‐alone simulations. This observation suggests differential stability even for highly similar structures. RMSF analysis confirms this observation as values are generally less than 3 Å, except for the disordered tail, which fluctuates extensively in the absence of the components of the complex (see Figure S11(A–C)).

**FIGURE 7:**
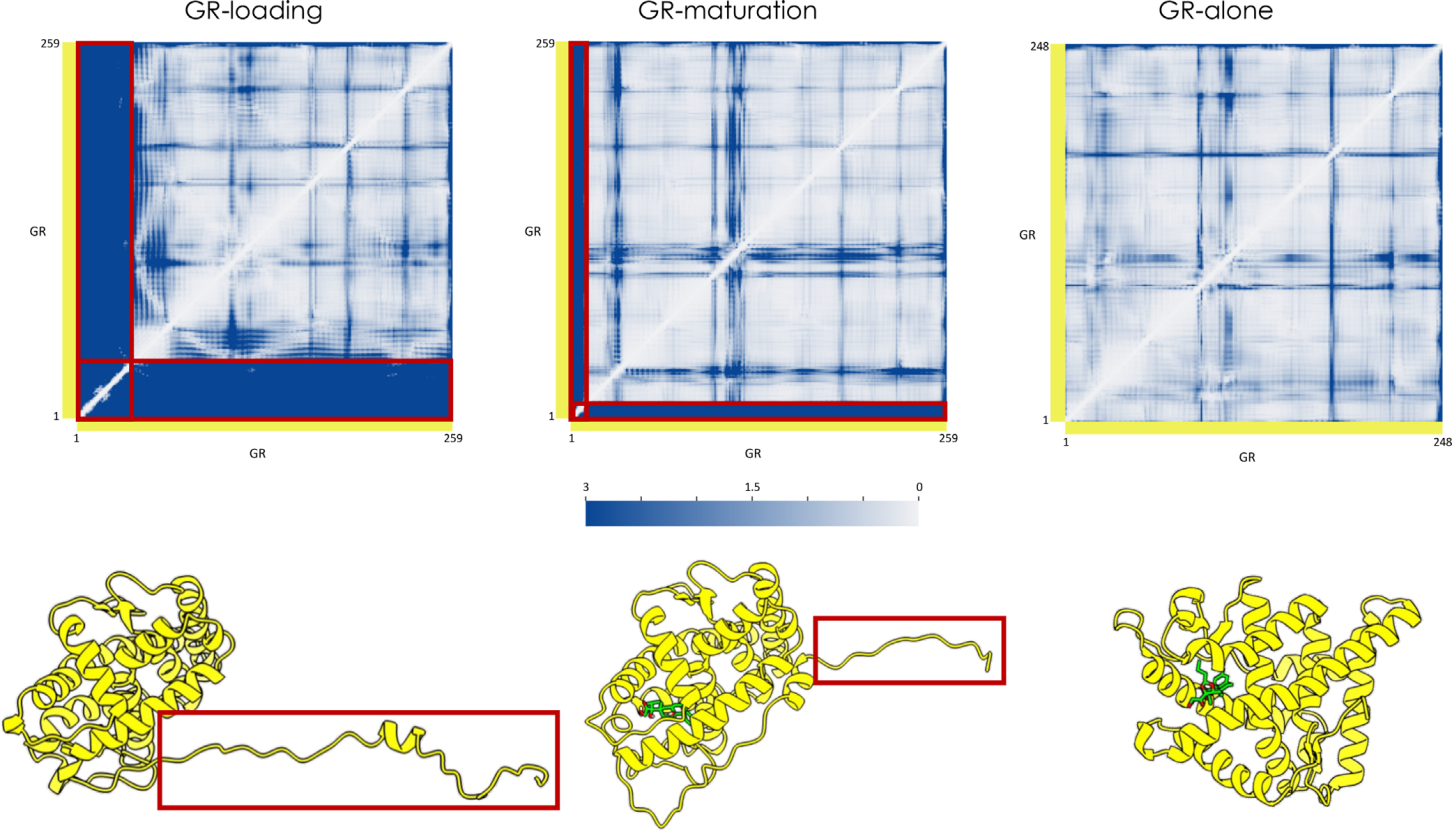
Residue‐pair distance fluctuation matrices for the client glucocorticoid receptor (GR) simulations See Table 1 for simulation labels; the axes report residue numbers. Top row: From left to right, the DF matrices for GR‐loading, GR‐maturation, and GR‐alone. Bottom row: The 3‐D structures of GR in the three different systems corresponding to the respective matrix in panel A. The red box in the matrices identifies the low coordination region of GR's tail; the corresponding region is highlighted in red boxes also on the respective 3‐D structure.

We also analyzed the internal dynamics features emerging from the simulations of GR starting from the three conformations described in the previous paragraph (Figure 7a). First, common shared coordination patterns for the folded core in the different states of the client are observed. As expected, the major differences reside in the unstructured tail residues of the conformations extracted from the protein complexes (Figure 7a and Figure 7b, red box). In particular, the GR_helix1_ and the GR_pre‐helix1_, that are partially folded and delineate the steroid‐binding site in GR‐Maturation, show rigid coordination with the folded core of the client as observed for the dynamics of GR‐alone, where the protein is in a fully native state. On the other hand, in the GR‐loading system, this region is not folded onto the protein and it appears to be highly flexible.

We then compared the internal dynamics of the client loaded onto the complexes (Loading‐ADP and Maturation) with the corresponding isolated ones (GR‐Loading and GR‐Maturation) (see Figure 7A and Figure S12). This analysis shows a general trend where the unstructured tail is rigidified when it is loaded onto the complexes. On the contrary, the folded core shows the same general pattern in the various simulated conditions. These data highlight the importance of the coordination with the binding sites defined by the large multimolecular complexes as well as their effects in modulating the dynamics of GR during the folding cycle.

### 2.7 The GR regions most prone to unfold in the native state are the ones engaged by the chaperone machinery

We set out to identify the regions of GR that are crucial for its stabilization and the ones that may be most prone to unfolding (Figure S13). In this framework, we analyzed the three simulations (GR‐Loading, GR‐Maturation, and GR‐alone) of the isolated version of the client and compared them against what was observed in the Loading‐ADP and Maturation‐ATP simulations (see Figure S13). To obtain the most representative conformational states of GR, we performed a clustering analysis (see *Methods* [Section 5]).

Next, we applied the matrix of local coupling energies (MLCE) analysis to the distinct cluster representatives. MLCE is a technique based on the analysis of the interaction energies of all the amino acid pairs in a protein. In particular, it computes the non‐bonded part of the potential (van der Waals, electrostatic interactions, solvent effects) via a MM/GBSA calculation, obtaining, for a protein composed by *N* residues, a *N × N* symmetric interaction matrix *M*_*ij*_. Eigenvalue decomposition of the matrix highlights the regions with the strongest and weakest couplings: the former indicates the stabilizing folding nucleus, while the latter defines the fragments that are on the surface, contiguous in space, and weakly coupled to the protein core and detect potentially unstable/unfolding regions that are poised to be engaged by interactions with the folding machinery (Morra & Colombo, 2008; Paladino et al., 2020; Scarabelli et al., 2010).

Interestingly, the regions of strongest couplings are fully conserved among all the analyzed states (residues 657–668, 704–705, and 714–718; UniProt Numbering), and define a shared folding core, characterized by a remarkable rigidity as shown in the aforementioned DF analysis (Figure 7C and Figure S13). Importantly, the rigid core is at the interface with Hsp90 protomer B in both the Loading‐ADP and Maturation‐ATP systems, and it is at the opposite side with respect to the GR active site. Moreover, the GR stabilization core is in direct contact with the GR_helix1_, which folds from the Loading to the Maturation Complex (GR_helix1_ slides and folds onto this part). This evidence supports the role of the identified stability core as a folding nucleus.

Finally, we identified the regions of native GR that are energetically minimally coupled to the folding core, representing potential unfolding substructures (Figure 7D). Importantly, we observed that one of the regions that are most prone to unfold in isolated GR is located at the interface between Hsp90 and GR in the Loading and Maturation Complexes. In particular, it comprises the GR terminal tail that contains the alpha helix seen to fold onto the stabilized nucleus of GR by sliding into the client site in the passage from the Loading to the Maturation stage. This result corroborates previous observations indicating that it is the tendency of substrate regions to undergo unfolding fluctuations that determines the client's engagement by the chaperone machinery.

### 2.8 Identification of potential druggable pockets from the dynamics of whole complexes

**FIGURE 8:**
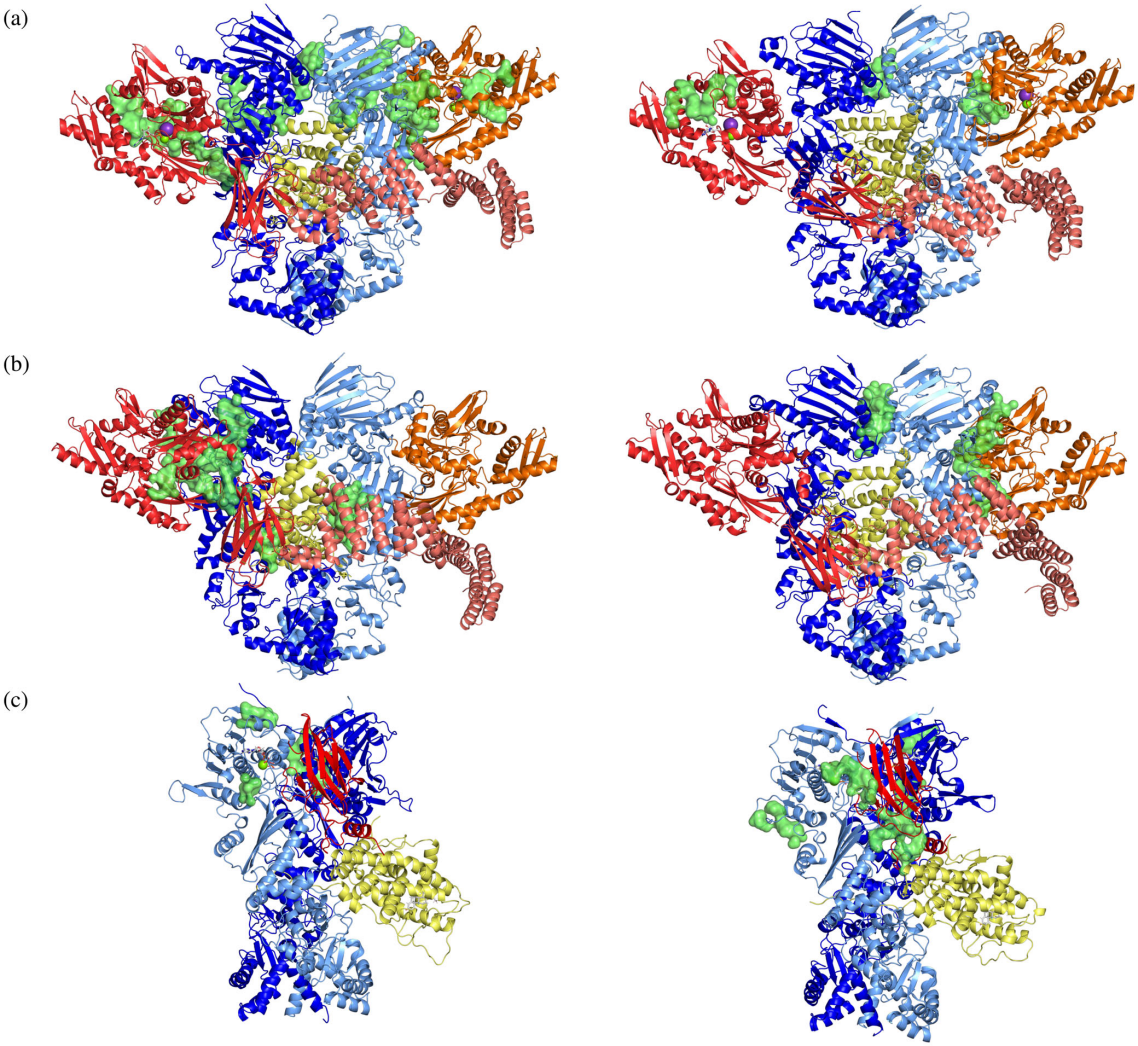
Potential druggable pockets to interfere with allosteric pathways. Identified druggable pockets are represented as green surfaces. (A) From left to right the structures of the most populated and the second most populated clusters from the trajectory of the Loading‐ADP. The green surfaces display pockets located along allosteric long‐range coordination pathways. (B) From left to right the structures of the most populated and the second most populated clusters from the trajectory of the Loading‐APO. The green surfaces display pockets located along allosteric long‐range coordination pathways. (C) From left to right the structures of the most populated and the second most populated clusters from the trajectory of the Maturation. The green surfaces display pockets located along allosteric long‐range coordination pathways.

The last analysis we carried out is a pocket search on the structures of the Loading‐ADP, Loading‐APO, and Maturation protein systems. Using the *Schrödinger*'s *Maestro* tool *SiteMap*, we looked for potential druggable pockets in the most representative structures from clusters retrieved from MD simulations. This analysis highlighted several regions, in diverse substructures of the distinct complexes, that could be actionable for drug design. To identify druggable pockets, we first ran the algorithm *SiteMap* on the whole assembly represented in a certain structural cluster (see *Methods* [Section 5]). Next, we filtered out and deemed as potential allosteric drug targets only the pockets that overlap with or belong to regions that are highly long‐range coordinated as determined by the above‐reported analyses of allosteric networks. Among these, we specifically focused on the pockets in regions that are highly coordinated with GR. Our working hypothesis is that once a ligand engages such pockets, it could interfere with GR folding in a specific and effective manner. For each system, the most significant pockets identified are reported in Figure 8.

Importantly, this screening is initially validated by independent findings: indeed we identified pockets that were already exploited for ligand design in completely independent and uncorrelated drug‐design initiatives. In this context, Hsp70C in the Loading‐ADP system was shown to play a key role in GR remodeling. The pockets identified with our approach on Hsp70C‐NBD indeed correspond to those identified in two previous studies, which were in turn used to develop biologically effective allosteric modulator YK5 (Rodina et al., 2013) and MKT‐007 (Rousaki et al., 2011) (see Table S1). Additionally, a druggable pocket was identified at the border between the Large and Small subunits of the Middle‐Domain of Hsp90 (Figure 8b) (See Table S1). This was previously used to develop allosteric ligands (D'Annessa et al., 2017) (Merfeld et al., 2023) with interesting pharmacological profiles.

These first qualitative validations support the feasibility of using structural and dynamic information from the simulation of large functional assemblies to guide the selection of novel leads.

## 3 DISCUSSION

In this study, we set out to provide atomic‐level understanding of the mechanisms of regulation and interaction in the large chaperone assemblies, namely the Loading and Maturation Complexes, that oversee folding and activation of the stringent client GR. We combined extensive equilibrium MD simulations with out‐of‐equilibrium simulations to shed light on the determinants of nucleotide‐dependent internal dynamic regulation and on the mechanisms of relay of conformational signals among the various components. We also investigated the origins of the detrimental effects on client folding of the perturbation of co‐chaperone Hop as well as the tendency of isolated GR to undergo unfolding in specific regions. Finally, we leveraged simulation results to identify potential binding pockets on the whole complexes, some of which interestingly correspond to regions that were proven to bind effective allosteric modulators of Hsp70/Hsp90 functions (D'Annessa et al., 2017; Merfeld et al., 2023; Rodina et al., 2013; Rousaki et al., 2011).

Investigation of the Loading Complex unveiled the importance of the highly intertwined among the dynamics of the various partners necessary to warrant correct functioning of the Loading Complex. As nucleotides are bound to the two Hsp70 active sites, extensive dynamic connection is seen among all the partners in the complex. Together, Hop–Hsp90 and Hsp70–Hsp90 interactions combined with the extensive coordinated dynamics, in particular at the interaction surfaces, determine the Hsp90 states that adapt to unfolded GR and are not yet ready to carry out clamp closure, ATP processing, and thus client remodeling. The resulting peculiar semi‐open conformation of Hsp90 solved in the Loading Complex indicates that this chaperone functions as an actual scaffolding protein, which, together with Hsp70 and Hop, forms the extensive interface that hosts the GR's unfolded tail. Interestingly, the unfolded GR region is highly coordinated with all the domains of the assembly, except for Hsp90 NTDs. The end task of the interface is to keep the unfolded GR subregion strictly in place and constrain its dynamics to avoid uncontrolled release, and at the same time slow down GR‐conformational rearrangements.

Interestingly, Y‐phosphorylation or mutations in Hop turn out to impact on these finely tuned networks, with clear reverberations on the dynamics of the whole complex and a key loss of coordination with the unfolded region of the client, consistent with experimental findings showing decreased GR activation upon Hop phosphorylation (Bhattacharya et al., 2020; Bhattacharya & Picard, 2021; Castelli et al., 2023).

Importantly, the release of the nucleotide from Hsp70 active sites increases the overall flexibility and causes a marked loss of internal coordination within and among the different partners of the assembly. D‐NEMD simulations highlight a conformational pathway whereby the signal encoded by ADP removal reaches the client through Hsp90_NTD_/Hsp70_NBD_ interfaces. In this model, these interfaces may act as checkpoint for the progression from the loading to the maturation complex (see Figures 3 and 4).

The interactions and dynamics in the Loading complex favor the semi‐open Hsp90 conformation, which is favorably poised for ATP binding in the NTDs and for a nucleotide dependent cross‐talk with the client. Indeed, the observed efficient communication between Hsp90 NTDs and the client binding site would expectedly facilitate the transduction of signal encoded by ATP‐hydrolysis to client remodeling. In this context, the coincidental increase in internal flexibility and loss of ordered dynamics upon Hsp70 nucleotide unbinding in the Loading Complex can be considered as an effective mechanism to make the whole complex less stable, favoring the onset of disassembly and transmission of GR to the next (maturation) step. Hsp90 is the unifying element, which undergoes significant structural and dynamical remodeling to populate an ATPase‐competent state.

Overall, not only does the Loading Complex provide a structural platform to bind the unfolded region of GR (or other client proteins), but its highly dynamic, adaptable states combined with their unique long‐range responsiveness to the nucleotide define an efficient machinery for client remodeling. The events at chaperone active sites are thus coupled to the functional cell requirements of sequestering a client unfolded state.

In the Maturation complex, GR, which is threaded through the lumen of Hsp90, adopts a more native‐like conformation and is experimentally seen to bind a steroid substrate. Hsp90 is in the closed, catalytically competent conformation, previously observed for the chaperone both in isolation and in complex with a kinase client.

Interestingly, in this structure, the internal dynamics of Hsp90 NTDs and its coordination motifs with the client and co‐chaperone are markedly different from what is observed in the Loading Complex. In this framework, the ATP‐bound active sites of Hsp90 are characterized by high coordination with the dynamics of the DEX‐bound active site of GR, and a large fraction of the fully folded domain of the client (See Figures 5 and 6). Interestingly, the connection between the Hsp90 and client active sites is markedly asymmetric, with one protomer influencing the GR‐chaperone interface and the other acting more specifically on the client active site. The asymmetric control and reactivity of Hsp90 (Elnatan et al., 2017; Lavery et al., 2014; Moroni et al., 2018; Partridge et al., 2014) permits to expand the space of conformations presented to interaction with clients, favoring the engagement of diverse substrates, while supporting a fine regulation of the mechanisms of client remodeling in combination with co‐chaperones. p23 plays a double role in this mechanism: it modulates ATPase by directly influencing the dynamics of the NTDs and controls the internal dynamics of the whole client. In this framework, p23 contributes to pre‐organize GR in a near‐folded conformation that is poised to bind the steroid.

The Maturation Complex emerges as a finely regulated machine, whose key function appears to be the modulation of the dynamics of the partially folded GR‐active site. Coherent with the nature of the Loading Complex and the general properties observed for the chaperone machineries at the biochemical and biophysical level, the Maturation Complex is a dynamic assembly where protein–protein interactions (PPIs) are optimized to orchestrate an efficient communication between the enzymatic activity of Hsp90 and the ligand binding activity of the client. The co‐chaperone p23, through its terminal helix, stabilizes the client, while influencing the dynamic states of Hsp90 NTDs (Pricer et al., 2017; Verba & Agard, 2017).

This multiprotein complex facilitates GR protection from degradation and aggregation, while favoring its processing and sliding through the lumen to deliver an active client ready for biological function.

Finally, the analysis of isolated GR shows that the regions with the highest propensity to unfold starting from the native state are in fact the ones that turn out to be in contact with Hsp90/Hsp70 and remodeled in the two complexes. Combined with previous experiment‐based observations (Noddings et al., 2022), this indicates that rather than the overall stability of the client, the determinant for engagement by the chaperone machinery is the probability/tendency of partial/local unfolding fluctuations.

Other clients are seen to be unfolded at specific regions, and their available 3D structures in complex with Hsp90 (or Trap1, or Grp94) show the unfolding part threaded in the lumen of the chaperone (Verba & Agard, 2017). The delicate interplay between unfolding propensity, recognition by the chaperone machinery, and dynamic regulation by the chaperoning assemblies can be considered a general mechanism for the control of protein structural quality in cells. In this context, the specific outcomes for folding and function of different clients may be distinct (with inhibition vs. activation depending on cellular needs) and regulated by the engagement of different chaperones and co‐chaperones, supporting a deterministic client‐specific structural remodeling.

Our results here suggest that the general unifying mechanism of Hsp90‐complexes is that the protein–protein interactions underpinning their functions are dynamic, conformationally heterogeneous, and highly responsive to ligand state or mutation states. An intricate and delicate network of interactions and coordination pathways defines the functional profile of distinct assemblies, while the dynamic nature of the interactions points to a degree of fuzziness that supports the weak affinities and short‐lives observed for chaperone complexes (Pricer et al., 2017; Verba & Agard, 2017; Wu et al., 2019). This can aptly favor the client hand‐off between different complexes at different stages of the chaperone cycle, while facilitating the recruitment of the co‐chaperones necessary to finely regulate chaperone functions.

The inherently transient nature of the complexes and their conformational plasticity, combined with the requirements for the formation of correct PPIs and selection of dynamic states, represent an evolutionarily determined mechanism to ensure binding and remodeling of different partners at different points of cell life. As noted by Gestwicki and co‐workers, (Pricer et al., 2017) conformational heterogeneity enables the emergence of condition‐specific function and conveniently allows a single structural motif (the client‐binding site or co‐chaperone binding interfaces) to be used in multiple contexts.

Finally, we showed the possibility of exploiting this new knowledge on the mechanism of internal dynamic regulation of various complexes to unveil potential small‐molecule binding pockets (Colombo, 2023). The latter can be targeted to influence the mechanisms of GR folding and maturation, via the perturbation of the functional communication pathways that underlie functionally oriented motions in the different complexes. Interestingly, in support of this method, we were able to select two pockets on the NBD of Hsp70 that were already reported in the literature as being able to bind allosteric inhibitors. The novel pockets identified here constitute an interesting platform for the design of new regulators of chaperone mechanisms (D'Annessa et al., 2017; Merfeld et al., 2023; Rodina et al., 2013; Rousaki et al., 2011).

## 4 CONCLUSIONS

Our study advances the molecular understanding of the regulation of the internal dynamic mechanisms that underlie the functions of the Hsp90/Hsp70 chaperone machinery, a key player in a wide range of vital cellular processes. Dysregulation of chaperone function is often associated with tumorigenesis, inflammation, and neurodegeneration. Our study provides a general conceptual basis for analyzing functionally oriented aspects of the dynamics of new complexes that will be solved in the future. Our results also set the stage for drug discovery efforts to interfere with the conformationally heterogeneous interactions within the Hsp90/Hsp70 heterocomplexes: this in turn could offer novel pharmacological opportunities to identify potential (cryptic) druggable pockets in an assembly‐ and context‐dependent manner to target pathological conditions.

## 5 METHODS

### 5.1 Construction of solvated models for GR, and for the loading and maturation complexes

Prior to MD simulations, Cryo‐EM structures of the Loading and Maturation Complexes are subject to a series of preparatory steps.

The starting structure for all simulations of the Loading Complex, either *apo* or with ADP bound in the active site of each Hsp70 (henceforth Hsp70_APO_ and Hsp70_ADP_, respectively), is the Cryo‐EM structure (PDB: 7KW7). Both Hsp90ɑ protomers were resolved from Glu16 to Asp699 (UniProt P07900), except for the charged loop in each protomer (from residue 223 to residue 283). Missing Hsp90ɑ fragments were modeled based on previous simulations (Moroni et al., 2018; Rehn et al., 2016; Sanchez‐Martin et al., 2020). All other proteins were kept as experimentally resolved in the pdb. No extra residues were modeled at either terminus.

The starting structure for simulations of the Maturation Complex is the Cryo‐EM structure (PDB: 7KRJ). Missing Hsp90ɑ loop was again modeled based on previous simulations (Moroni et al., 2018, Rehn et al., 2016, Sanchez‐Martin et al., 2020) as for the Loading Complex; both protomers were thus reconstructed without gaps from Glu16 to Ile698 (UniProt P07900). The portion of GR's ligand‐binding domain resolved in this complex lacks four residues in its *N*‐terminus (SIVP), compared to the portion present in the Loading Complex: to make both systems comparable, these four residues were modeled into the lumen using the *PyMOL* molecular modeling package (Schrödinger, LLC. The *PyMOL* Molecular Graphics System, Version 1.8. 2015). GR was modeled from residue 519 to residue 777 (UniProt P04150) in both complexes. p23 was kept as experimentally resolved in the pdb. No extra residues were modeled at either terminus.

For each complex, the resulting structure was then preprocessed with *Schrödinger MAESTRO* suite (Schrödinger Release 2021‐1, Schrödinger, LLC, New York, NY, 2021): all residues were predicted by *PropKa* to be in their standard protonation states (Olsson et al., 2011). Histidine tautomer was predicted using the H‐bond optimization tool during protein preparation. Both pdb structures were confirmed not to feature any disulfide bridges. *AmberTools*' *tleap* utility (version 19) (Case et al., 2018) was used to fully protonate/deprotonate the predicted models. Finally, again with *tleap*, all models were solvated in a (periodic) cuboidal box of water, so that every atom in the complex was no closer than 11.0 Å to the nearest box edge, and an appropriate number of Na^+^ cations were randomly added as required to neutralize excess negative charge.

In addition, three independent simulations of the *N*‐terminal ligand binding domain of GR alone were conducted starting from three different states that are representative of its folding cycle. The first consists of the partially unfolded GR structure present in the Loading Complex. The structure was extracted from the relevant multiprotein complex and solvated with *tleap* using the same conditions used for the preparation of the other structures. Similarly, the active and competent state of GR present in the maturing complex, including the dexamethasone ligand, was considered and simulated. Finally, the structure of GR in the active free form (PDB: 5NFP), resolved from residue Pro 530 to Lys 777 (UniProt P04150), was prepared and simulated. The protein had its ligand removed and was solvated as in the other cases.

Topologies and atomic coordinates of all solvated starting structures are available as Supporting Material at https://drive.google.com/drive/folders/1‐sKIgqZGgCPj7an04XuSxy‐S8_Z6JDTu?usp=sharing.

### 5.2 Simulation parameters and MD simulations

All protein residues were modeled using the *ff14SB* forcefield (Maier et al., 2015). Water was described according to the TIP3P model (Jorgensen et al., 1983), and Na^+^ cations according to parameters published by Joung and Cheatham (Joung & Cheatham, 2008). Mg^2+^ was modeled according to parameters by Allnér and Nilsson (Allnér et al., 2012). ATP was treated using ad hoc parameters by Meagher and coworkers (Meagher et al., 2003). Forcefield parameters for GR ligand dexamethasone (DEX) present in the maturation complex were derived with the aid of *Gaussian16* package (Frisch et al., 2016) and of *AmberTools' antechamber* and *parmchk2*(Case et al., 2018; Wang et al., 2006): first, DEX was structurally optimized in the gas phase at the B3LYP/6‐31G(d) level of density functional theory (DFT); then, ESP charges (Lee et al., 1988) around each atom were calculated based on the electrostatic potential, calculated at the Hartree‐Fock/6‐31G(d) level, and sampled over 10 shells per atom at a density of 17 grid points per square Bohr; final atomic point charges were then assigned after RESP (Bayly et al., 1993) fitting, performed *by antechamber*, which assigned atom types were assigned according to the *gaff* force field (Wang et al., 2004).

All molecular mechanical molecular dynamics (MM MD) simulations featured in this work were conducted using the *Amber* molecular simulation suite (version 18) (Case et al., 2018), either employing the *sander* utility in the earliest preproduction stages or the *pmemd*.cuda utility (relying on GPU acceleration) otherwise (Salomon‐Ferrer et al., 2013). Four independent MD replicas (atomic velocities assigned from different random seeds) were carried out for each system (see Table 1), comprising pre‐minimization, minimization, heating, equilibration, and production.

Firstly, a *sander* pre‐minimization was carried out in order to relieve the strain generated by newly modeled fragments and to resolve any steric clashes. The systems were minimized with a two‐stage approach to remove any bad contacts between solute and solvent. In the first stage, a 300‐step minimization was carried out on all hydrogens using 10 steps of steepest descent algorithm, followed by 290 steps of conjugate gradient method. Positional restraints were introduced on all heavy atoms with a harmonic force constant equal to 5 kcal mol^−1^ Å^−2^. In the second stage, a 300‐step minimization was performed on the whole system without positional restraints and using the same combination of 10 steps of steepest descent method and 290 steps of conjugate gradient as in the first stage. Solvent equilibration was performed with a 9 ps simulation in the *NVT* ensemble (time step, 1 fs), assigning positional restraints (force constant of 10 kcal mol^−1^ Å^−2^) to all solute atoms. This process is divided into three steps: a first 3 ps heating from 25 to 400 K, followed by a 3 ps equilibration at the constant temperature of 400 K and a third cooling to 25 K in the remaining 3 ps. Temperatures are maintained by the Berendsen thermostat (Berendsen et al., 1984). A tight temperature coupling (0.2 ps) is set for the first 6 ps of the process and then relaxed during the 3 ps cooling phase (from 1.0 to 2.0 ps). The temperature of the whole system was then increased from 25 to 300 K through a 20 ps simulation (time step, 2 fs) in the *NVT* ensemble. In order to avoid any large fluctuations, a combination of harmonic positional restraint (applied to Cα atoms with a force constant of 5 kcal mol^−1^ Å^−2^) and Langevin thermostat (Loncharich et al., 1992) (collision frequency set at 0.75 ps^−1^) was used. The *SHAKE* algorithm is used to constrain hydrogen‐containing bonds (Miyamoto & Kollman, 1992).

Next, the system was equilibrated with a 3‐step protocol. All equilibration steps use an *NpT* ensemble, a 300 K temperature (controlled by the Langevin thermostat (Loncharich et al., 1992), collision frequency 1 ps^−1^), 1 atm pressure (controlled by the Berendsen barostat (Berendsen et al., 1984)) and the *SHAKE* algorithm (Miyamoto & Kollman, 1992). The whole process lasts 2.04 ns (time step, 2 fs). The first 20 ps of equilibration is performed by relaxing position restraints on backbone Cα to 3.75 kcal mol^−1^ Å^−2^. The second step further relaxes the position restraints to 1.75 kcal mol^−1^ Å^−2^ during 20 ps of simulations. The third step is 2 ns in length, and no backbone restraints are left active.

MD production was carried out with initial velocities for each replica obtained from a Maxwellian distribution at the initial temperature of 300 K (to ensure the independence of each replica). The temperature was kept constant with the Langevin thermostat (collision frequency of 5.0 ps^−1^) (Loncharich et al., 1992) and a constant pressure of 1 bar was introduced via Berendsen's barostat (2 ps relaxation time) (Berendsen et al., 1984). Electrostatic interactions were treated using the particle mesh Ewald method (Darden et al., 1993) with a cutoff of 8 Å. The same cutoff was used even for short‐range Lennard‐Jones interactions. The *SHAKE* algorithm is used to constrain bonds containing hydrogen. Production runs of each replica were extended to 1 μs in the *NpT* ensemble (time step, 2 fs), with an identical setup to the final equilibration condition.

Postprocessing and analysis of MD trajectories were conducted with the CPPTRAJ tool (Roe & Cheatham III, 2018) distributed within the *AmberTools* suite (version 19) (Case et al., 2018), whereas the VMD platform was used for visual inspection (Humphrey et al., 1996).

### 5.3 Trajectory analysis

Root‐mean‐square‐deviation *(RMSD)* of the backbone atoms with respect to starting Cryo‐EM structures was computed aligning on the backbone atoms using the *rmsd* command implemented in the CPPTRAJ program distributed within the *AmberTools* suite (version 19) (Case et al., 2018). Calculations were carried out independently for each system (see Table 1).

Root‐mean‐square‐fluctuation values *(RMSF)* were computed on a per‐residue basis using the *atomicfluct* command implemented in CPPTRAJ (Roe & Cheatham III, 2018): as a reference for *RMSF* calculations, we employed the average structure in the MD trajectory being analyzed.

### 5.4 Clustering

Cluster analysis was performed for each of the simulated systems using the hierarchical agglomerative algorithm from the *cluster* command in CPPTRAJ (Roe & Cheatham III, 2018). Clustering was carried out as follows: first, all independent MD replicas for each system under study were concatenated into metatrajectories, with backbone heavy atoms of ɑ‐helices and β‐sheets in each frame aligned onto corresponding atoms in the system's starting structure. Then, clustering itself is performed based on the RMSD of backbone heavy atoms of all remaining (disordered) regions, thus singling out individual conformational families with unstructured regions that are as conformationally diverse as possible. For each simulated system (see Table 1), the number of clusters was selected through silhouette analysis.

For Loading‐ADP and maturation systems, six representative conformations were collected. GR‐alone simulations were clustered in two representative structure, while for GR‐Loading and GR‐Maturation we identified three clusters each.

### 5.5 Distance fluctuation analysis

Distance fluctuations (DFs) to uncover potential allosteric communication pathways were calculated after combining the four MD production replicates for each simulated system into a single 4 μs metatrajectories aligned on C_α_. Individual elements *DF*_*ij*_ of the DF matrix **DF** corresponds to the distance fluctuation between a variant's *i* ^th^ and *j* ^th^ amino acid, and are calculated as follows:

$${\mathrm{DF}}_{\mathrm{ij}}=\left\langle {\left(d_{\mathrm{ij}}-\left\langle d_{\mathrm{ij}}\right\rangle \right)}^{2}\right\rangle ,$$

where *d*_*ij*_ denotes the distance between C_α_ atoms of amino acids *i* and *j* in a given metatrajectory frame, and quantities expressed between ⟨⟩ are averages over all frames in a system's metatrajectory. The higher the DF for a residue pair, the less quasi‐rigidly it moves (low allosteric coordination); vice versa, low DF scores denote residue pairs moving in a highly coordinated manner (Moroni et al., 2018; Morra et al., 2012; Rinaldi et al., 2018). DF analysis in this work was conducted using our ad hoc code (available at: https://github.com/colombolab/Distance-Fluctuation-DF-Analysis).

In order to simplify the visualization of the full (pairwise) DF matrices of the Maturation, the Loading‐ADP, and the Loading‐APO systems, we provide a simplified version of the matrices in which average “blockwise” DF scores are calculated after grouping individual residues on the *x* and *y* axes in blocks of 40 × 40 (i.e., in which each block contains average DF scores for 1600 residue pairs), and rounding these average DF scores, so that there are only five shades of white‐blue.

DF PyMOL movies for all the simulations reported in Table 1 are electronically provided at https://drive.google.com/drive/folders/1-sKIgqZGgCPj7an04XuSxy-S8_Z6JDTu?usp=sharing. For further information, a README.txt file is available.

### 5.6 Energy coupling

MLCE (Matrix of local coupling energies) is a technique analysis interaction energies of all the amino acids in a protein (Genoni et al., 2012; Morra et al., 2014; Morra & Colombo, 2008; Paladino et al., 2015; Scarabelli et al., 2010). In particular, after an initial minimization of chosen clusters using *sander* (Case et al., 2018), it calls *AmberTools'* MMPBSA.py tool (Miller et al., 2012) to compute the non‐bonded part of the potential (van der Waals, electrostatic interactions, solvent effects) via a MM/GBSA calculation, obtaining, for a protein composed by N residues, a N×N symmetric interaction matrix **M** with *N* ^2^ elements $M_{\mathrm{ij}}$. This matrix can be expressed in terms of its eigenvalues and eigenvectors (diagonalized) as:

$$M_{\mathrm{ij}}=\sum_{\alpha =1}^{N}\lambda_{\alpha }\upsilon_{i}^{\alpha }\upsilon_{j}^{\alpha },$$

where $\lambda_{\alpha }$ is the *α*‐th eigenvalue and $\upsilon_{i}^{\alpha }$ is the *i*‐th component of the corresponding eigenvector. The eigenvector with the most negative correspondent eigenvalue contains most information for the most stabilizing interaction of the system. On top of the most negative eigenvalue and its associated eigenvector, Genoni et al. have established that selecting additional eigenvectors that recapitulate those specific subsets of interactions that define possible folding nuclei within a protein structure (Genoni et al., 2012). An approximated interaction matrix $\tilde{M}$ with elements *M*_*ij*_ is thus given by only summing these more important eigenvectors/eigenvalues.

If the structure of the protein is known, one can, in parallel, estimate a residue‐residue contact matrix ***C*** whose elements *C*_*ij*_ are 0 if Cɑ atoms of residue pair *i‐j* are farther than 6 Å; or 1 otherwise. The Hadamard product of matrices $\tilde{M}$ and ***C*** then gives the Matrix of the Local Coupling Energies MLCE:

$${\text{MLCE}}_{\mathrm{ij}}={\tilde{M}}_{\mathrm{ij}}\times C_{\mathrm{ij}},$$

which will have nonzero elements MLCE_*ij*_ (equal to *M*_*ij*_) only if residues *i* and *j* spatially close enough.

Contiguous residues showing the weakest interactions are considered to be most prone to form interfaces with other proteins.

### 5.7 Dynamical non‐equilibrium molecular dynamics ( *D‐NEMD* ) simulations of the loading complex

To characterize the allosterically driven conformational changes and study the communication pathways within the multiprotein complexes, we performed a large number (176) of short (20 ns) non‐equilibrium MD simulations. Specifically, we applied the *D‐NEMD* methodology used successfully in previous studies (Oliveira et al., 2019; Oliveira et al., 2021) and first proposed by Ciccotti et al. in conjunction with the subtraction method.

**SCHEME 1:**
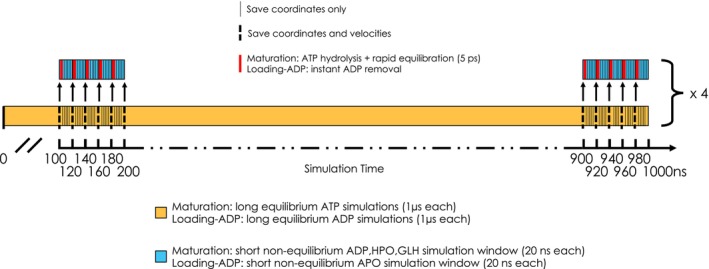
Pictorial scheme of the implementation of the D‐NEMD approach.

From the equilibrated part of each of the four 1 μs‐long MD replicas (from 100 ns to the end), conformations were extracted every 20 ns, for a total of 176 (44 per replica). In each of these 176 conformations, ADP molecules were deleted from Hsp70S_NBD_ and Hsp70C_NBD_. For the 176 resulting *apo* structures, we reinitiated as many short Hsp70_APO‐D‐NEMD_ MD simulations (20 ns each), maintaining the same velocities of the selected frame. To quantify the effect of the introduced perturbation (ADP removal) on every single residue in the Loading Complex, we employed the Kubo Onsager relation, whereby the short‐term response of the system to the introduced perturbation is calculated by averaging, over all 176 cases, positional deviations of each residue's C_α_ between perturbed (Hsp70_APO_) and unperturbed MD simulations at equivalent points in time (for a schematic representation of the D‐NEMD implementation see Scheme 1). This approach minimizes the noise from the intrinsic fluctuations of the systems (effectively canceling out natural movements in the most flexible regions), with the idea that any remaining deviations in the first few picoseconds—whether large or small—are directly relatable to the perturbation itself.

### 5.8 *D‐NEMD* simulations of the maturation complex

The *D‐NEMD* approach was also employed to study the effects of ATP hydrolysis in the maturation complex. As in the Loading Complex case, from the four trajectories we extracted conformations of the system every 20 ns, discarding the first 100 ns and obtaining 44 structures for each replica (176 in total). For each conformation, the relevant post‐hydrolysis state (consisting of ADP, HPO, and GLH47; for HPO [H_2_PO_4_]^2−^ we introduced parameters by Kashefolgheta and Vila‐Verde (Kashefolgheta et al., 2017)) was manually constructed, alternately mimicking ATP hydrolysis in the active site of protomer A and protomer B, then producing 352 starting structures for the non‐equilibrium MD simulations. All atoms displaced to generate the reaction products were placed to reproduce conformations compatible with the equilibrium conditions of the force field, and they were assigned the same velocities they possessed at the time of conformation extraction. We simulated all the hydrolyzed systems for a time of 20 ns by restoring the *SHAKE* algorithm (time step 2 fs). Applying the same method used to calculate the C_α_ deviation of the Loading Complex (for a schematic representation of the D‐NEMD implementation see Scheme 1), we can observe the effects on the structure and dynamics of the system due exclusively to hydrolysis of the nucleotide at one of the active sites. At the end of this procedure, a total of 174 simulations were completed and used for protomer A and 157 for protomer B.

### 5.9 Pocket search

Pockets were searched using the *SiteMap* module from *Schrödinger*'s *Maestro* suite. *SiteMap* allows identifying possible binding pockets inside a protein (Halgren, 2007; Halgren, 2009). The pocket search was carried out on the structural representatives of the two most populated clusters of the Loading‐ADP, Loading‐APO, and Maturation systems, covering more than 50% of the conformational space for each model. *Sitemap*, in addition to identifying binding regions, also assigns a druggability score to pockets (0 to 1). Of the 25 sites returned by the tool, only those having a score above 0.8 were considered and reported in Figure 8 (which are considered as most druggable).

## AUTHOR CONTRIBUTIONS

**Giorgio Colombo:** Conceptualization; investigation; funding acquisition; writing – original draft; methodology; validation; visualization; project administration; resources. **Matteo Castelli:** Investigation; methodology; validation; visualization; writing – original draft. **Andrea Magni:** Investigation; visualization; validation; methodology; writing – original draft. **Giorgio Bonollo:** Investigation; validation. **Silvia Pavoni:** Writing – original draft; resources. **Francesco Frigerio:** Writing – original draft; resources. **A. Sofia F. Oliveira:** Investigation; writing – original draft; methodology. **Fabrizio Cinquini:** Methodology; resources. **Stefano A. Serapian:** Writing – original draft; methodology.

## Supporting information


**Data S1.** Supporting Information.
