# Supplementary material for: Molecular mechanisms of chaperone‐directed protein folding: Insights from atomistic simulations
Source: Protein Sci. 2024 Feb 26;33(3):e4880. doi: 10.1002/pro.4880 (PMC10895457; doi:10.1002/pro.4880)
Supplement: Supplementary file 1 — Data S1. Supporting Information. [file PRO-33-e4880-s001.pdf]

SUPPORTING INFORMATION

Molecular Mechanisms of Chaperone Directed Protein Folding: Insights from Atomistic Simulations

Matteo Castelli,[1,#] Andrea Magni,[1,#] Giorgio Bonollo,[1] Silvia Pavoni,[2] Francesco Frigerio,[2] Fabrizio Cinquini,[3] Stefano A. Serapian,[1] Giorgio Colombo[1]*

1) Dipartimento di Chimica, Università di Pavia, via Taramelli 12, 27100 Pavia (Italy)

2) Department of Physical Chemistry, R&D Eni SpA, via Maritano 27, 20097 San Donato Milanese (Mi), Italy

3) Upstream & Technical Services – TECS/STES – Eni Spa, via Emilia 1, 20097 San Donato Milanese (Mi), Italy

# These authors contributed equally to this manuscript

* Author to whom correspondence should be addressed: g.colombo@unipv.it

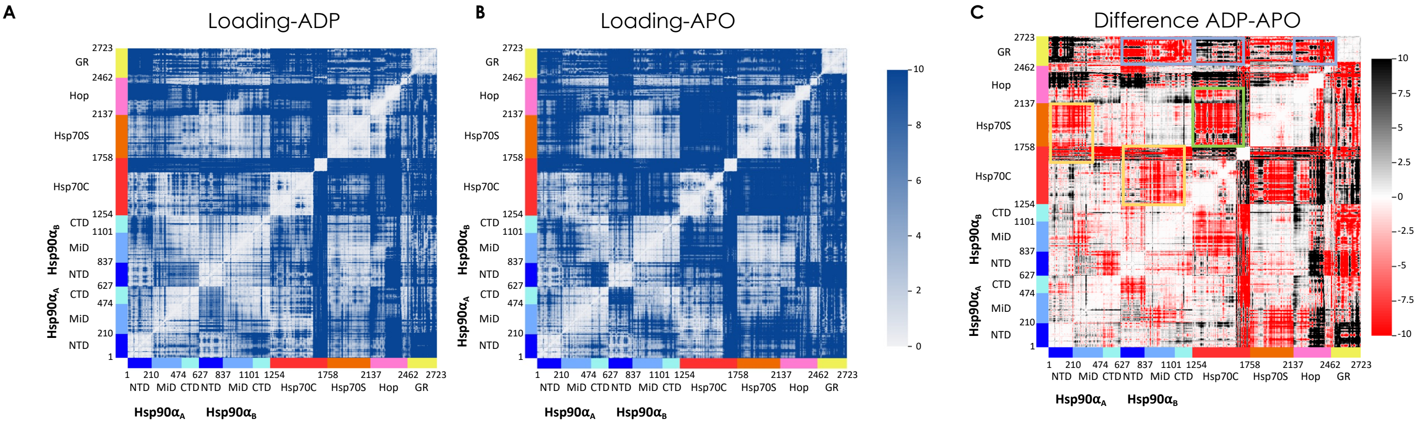

Figure S1. Full-detail Residue-Pair Distance fluctuations matrix of the **(A)** Loading-ADP system and of the **(B)** Loading-APO system. Here, lighter pixels correspond to highly coordinated residue pairs, while darker ones report on low coordination pairs. **(C)** DF difference matrix between the Loading-ADP matrix and the Loading-APO matrix. The axes report the residue numbers and the protein/domain division. In the difference matrix red pixels represent a more rigid coordination of the Loading-ADP pair residue respect to the Loading-APO pair residue, while the black pixels represent a less coordination of the Loading-ADP pair residue respect to the Loading-APO pair residue. The white pixels represent the same coordination, instead. Coloured boxes in panel **C** refer to Figure 3 (C–E) in main text.

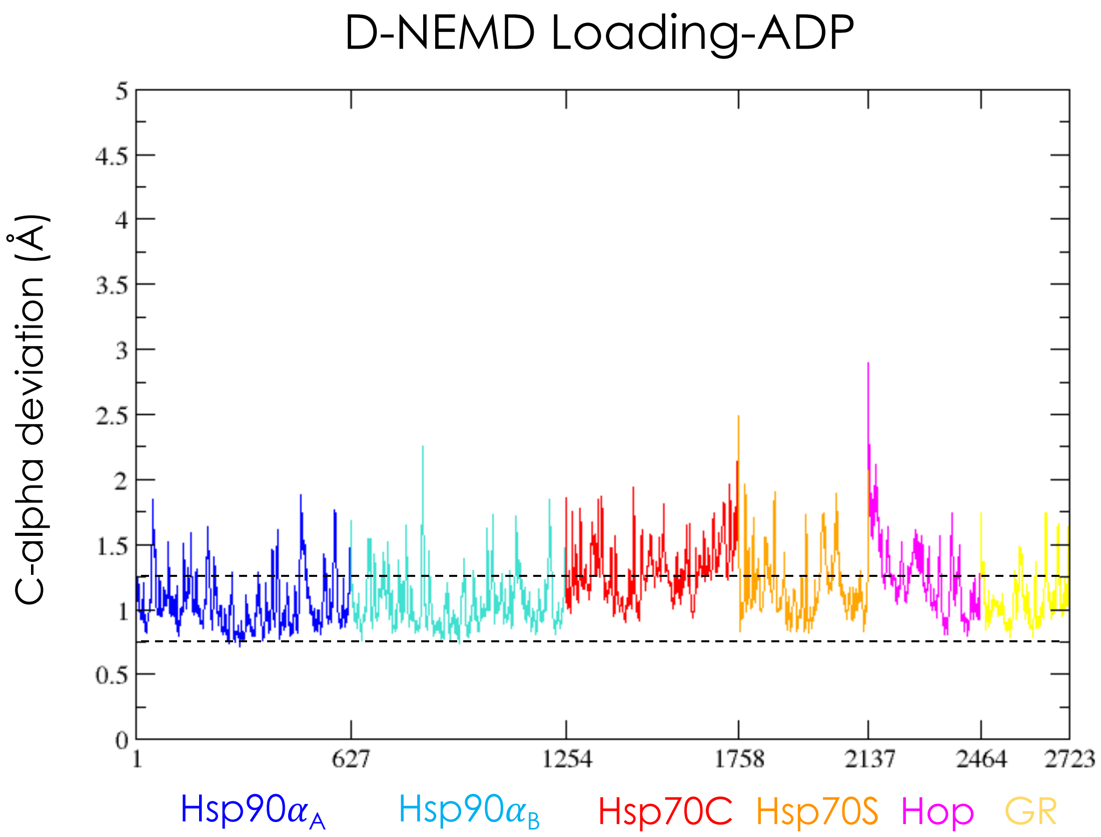

Figure S2. Average Cα deviation (Å) for each residues between the equilibrium Loading-ADP simulations and the non-equilibrium APO simulations. The average deviation is obtained from the 176 non-equilibrium simulations windows at 5 ns after ADP removal (see *Method* for details). On the x-axis are reported the single components of the multiprotein complex (the numeration corresponds to the one used in our simulations).

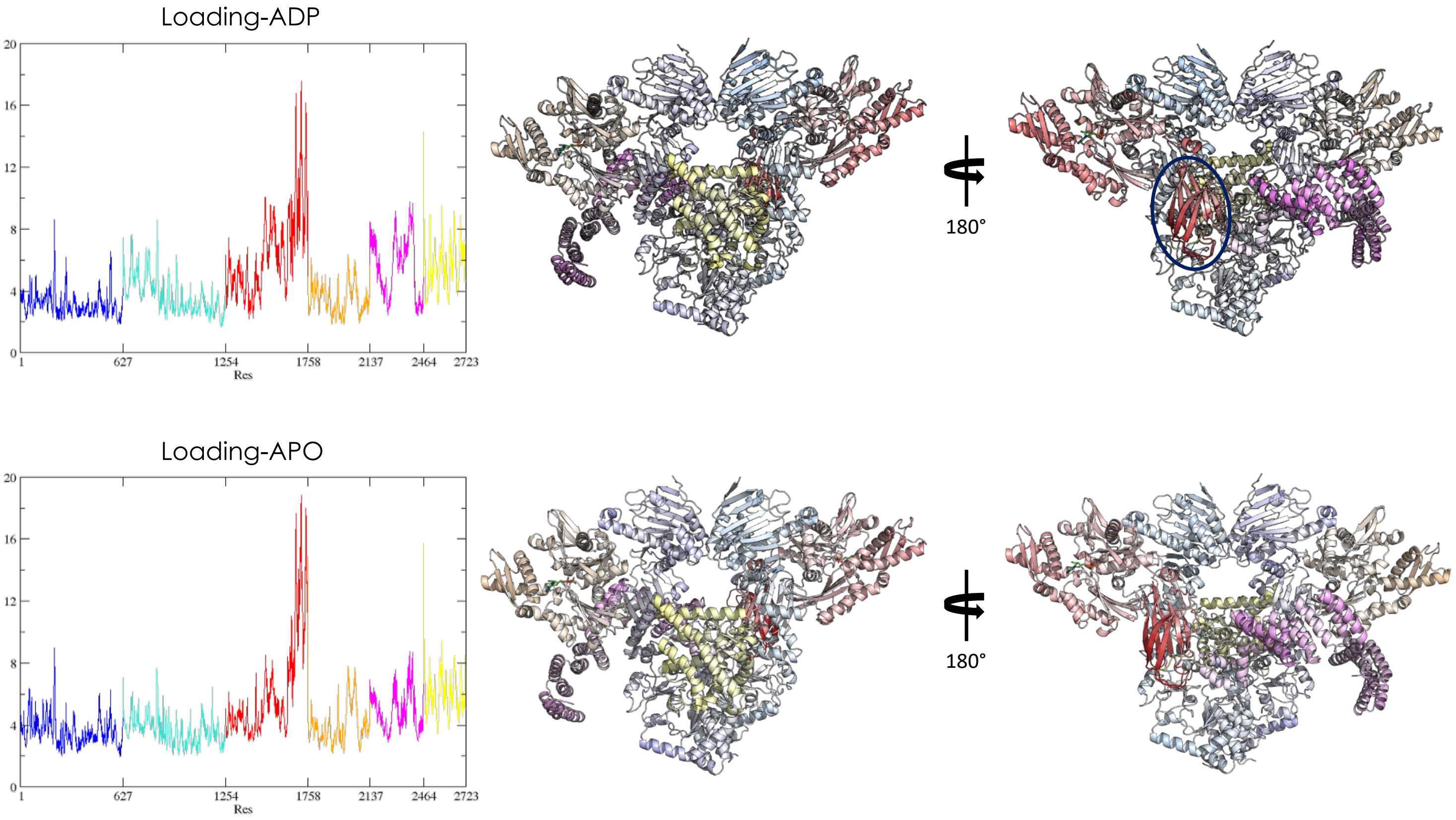

Figure S3. In the left portion of the picture there are the RMSF plots of the whole trajectories. In detail, the upper plot refers to Loading-ADP system, while the lower refers to the Loading-APO. The right portion of the picture contains the 3D structures of the loading complex, on which RMSF values are projected. Higher is the color intensity, higher is the RMSF value. The upper representations refer to the front and rear view of the loading complex in the Loading-ADP simulations, while the lower respresentations refer to the same views of the loading complex, but in the Loading-APO simulations, instead.

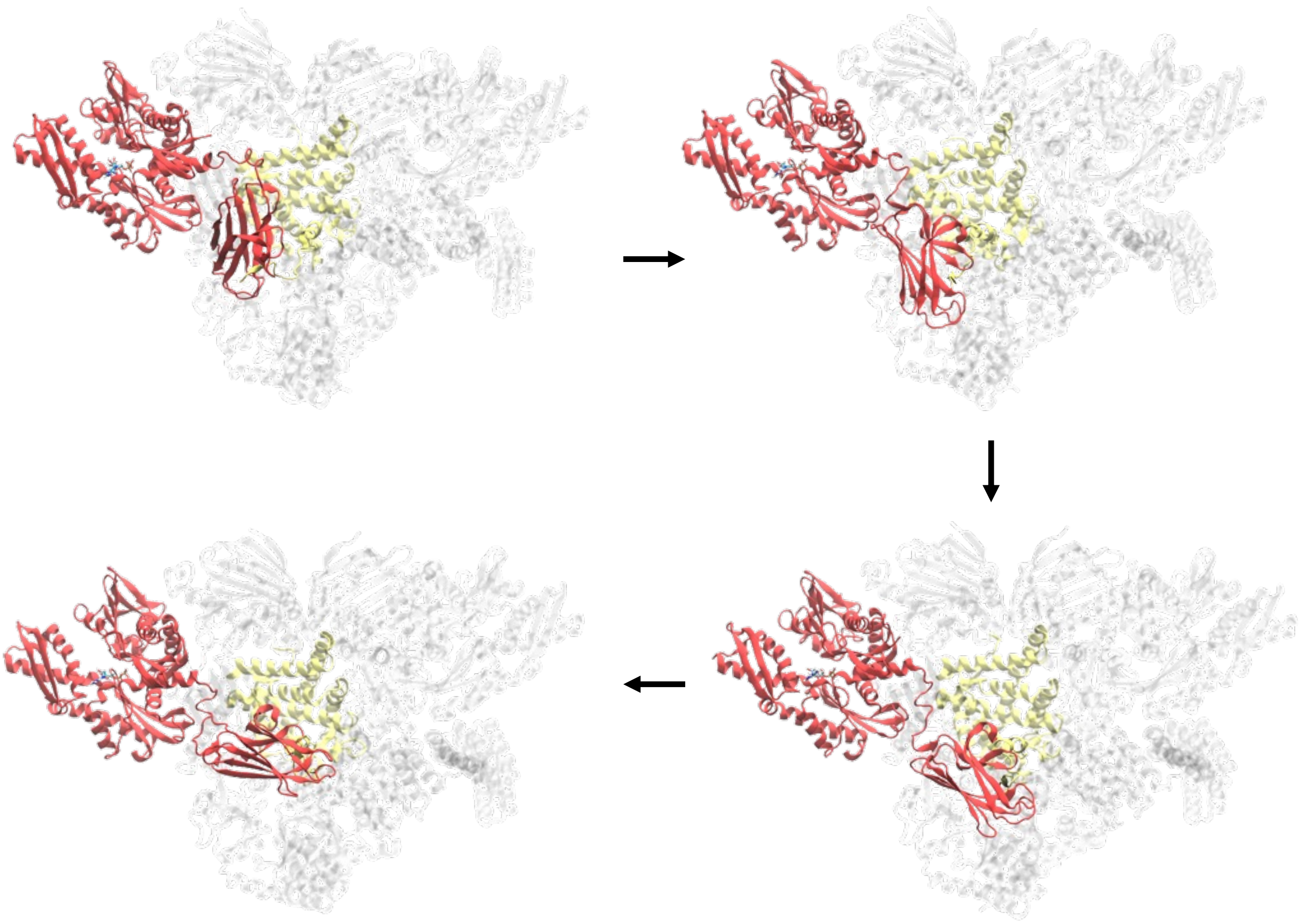

Figure S4. In the image sequence, from left to right, it is represented the Hsp70C$_{SBD}$ and GR tail movement that occurs during the simulations of the Loading-ADP.

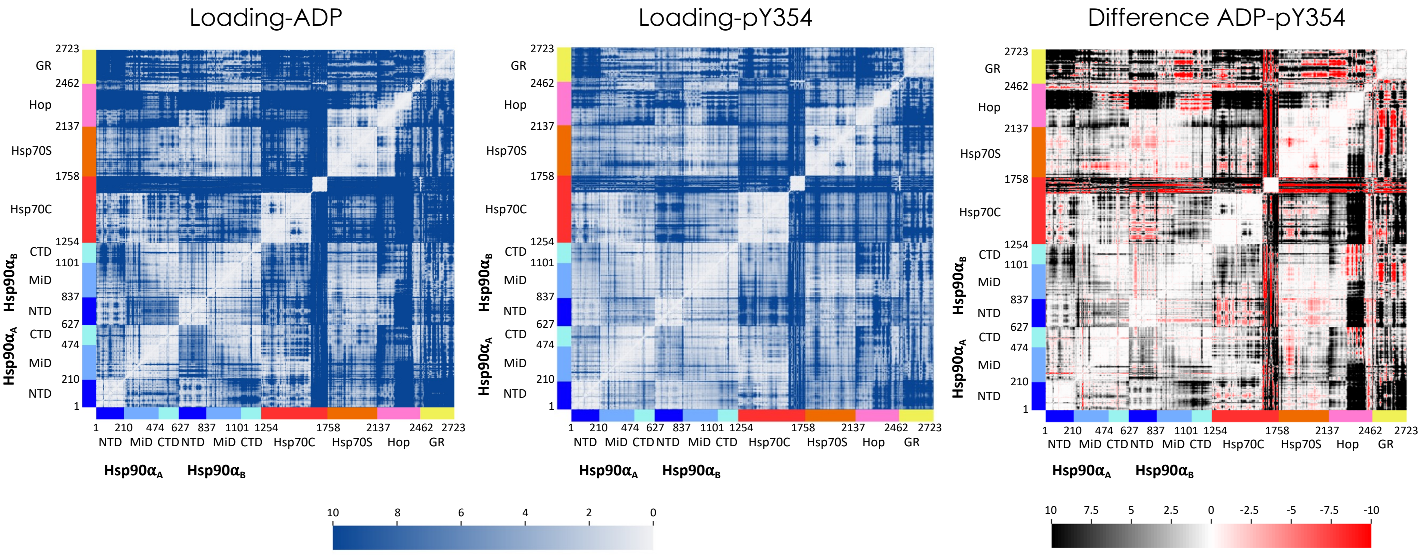

Figure S5. Distance Fluctuations matrices of the Loading-ADP system and of the Loading-pY354 system. The last matrix on the right represents the pairwise residue difference matrix between the Loading-ADP DF matrix and the Loading-pY354 DF one. On the axes there are the residue numbers (sequence) and the protein/domain division. The difference matrix has a colour bar that goes from red to black. Red coloured pixels represent a more rigid coordination of the Loading-ADP pair residue respect to the Loading-pY354 pair residue, while the black pixels represent a less coordination of the Loading-ADP pair residue respect to the Loading-pY354 pair residue. The white pixels represent the same coordination rigidity, instead.

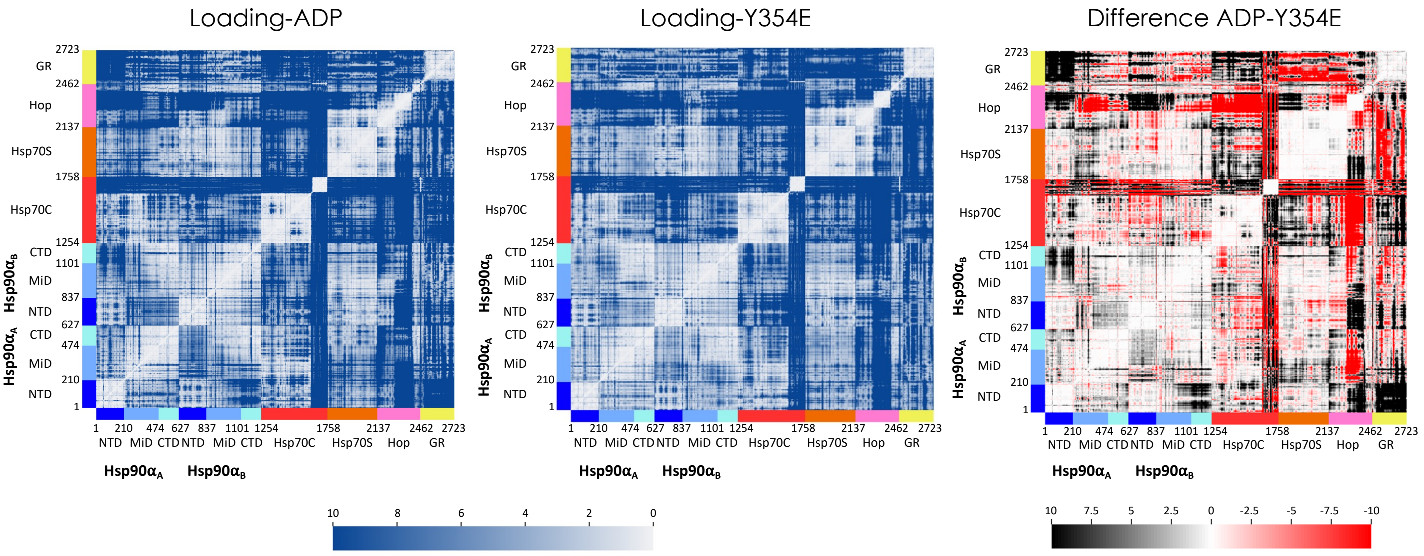

Figure S6. Distance Fluctuations matrices of the Loading-ADP system and of the Loading-Y354E system. The last matrix on the right represents the pairwise redidue difference matrix between the Loading-ADP DF matrix and the Loading-Y354E DF one. On the axes there are the residue numbers (sequence) and the protein/domain division. The difference matrix has a color bar that goes from red to black. Red colored pixels represent a more rigid coordination of the Loading-ADP pair residue respect to the Loading-Y354E pair residue, while the black pixels represent a less coordination of the Loading-ADP pair residue respect to the Loading-Y354E pair residue. The white pixels represent the same coordination rigidity, instead.

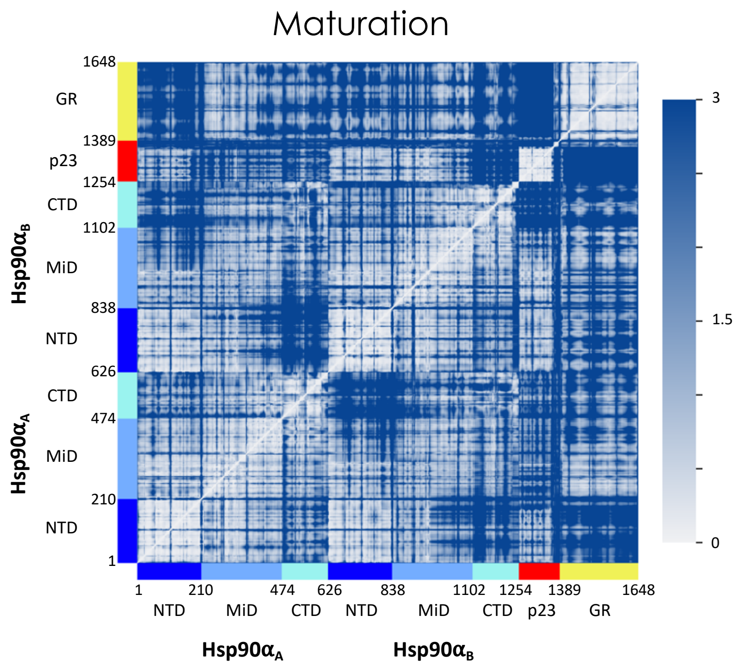

Figure S7. Full Distance Fluctuations matrix of the Maturation system (see Table 1 for simulation labels). Here, lighter pixels correspond to highly coordinated residue pairs, while darker ones report on low coordination pairs. On the axes there are the residue numbers (sequence) and the protein/domain division.

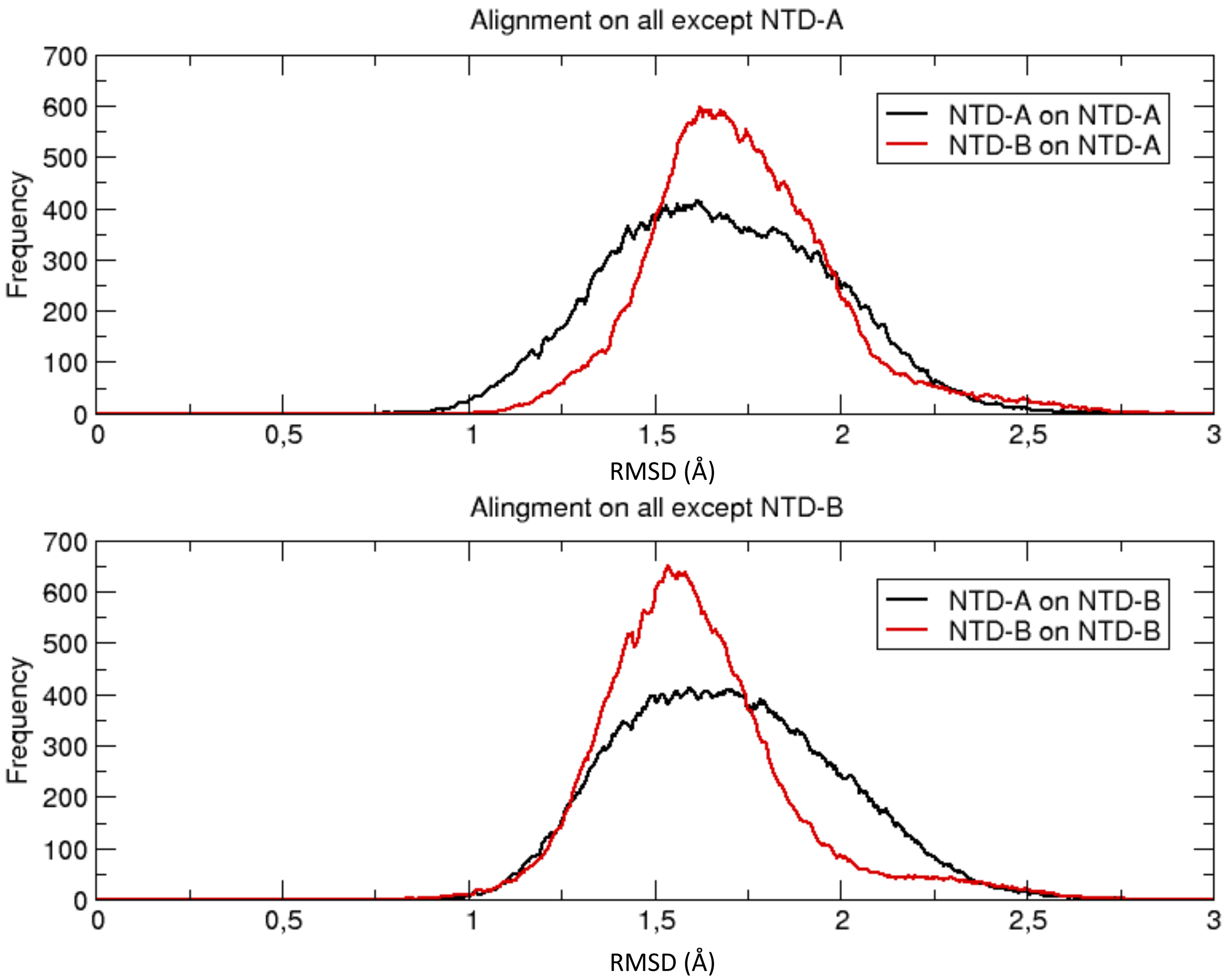

Figure S8. RMSD plots referred to the Maturation system simulations. In detail, the upper plot represent the RMSD calculated on the NTD-A (black) and NTD-B (red) of Hsp90 when the trajectory is aligned on the protein backbone of Hsp90 except for NTD-A. The bottom plot, instead, represent the RMSD calculated on the NTD-A (black) and NTD-B (red) of Hsp90 when the trajectory is aligned on the protein backbone of Hsp90 except for NTD-B.

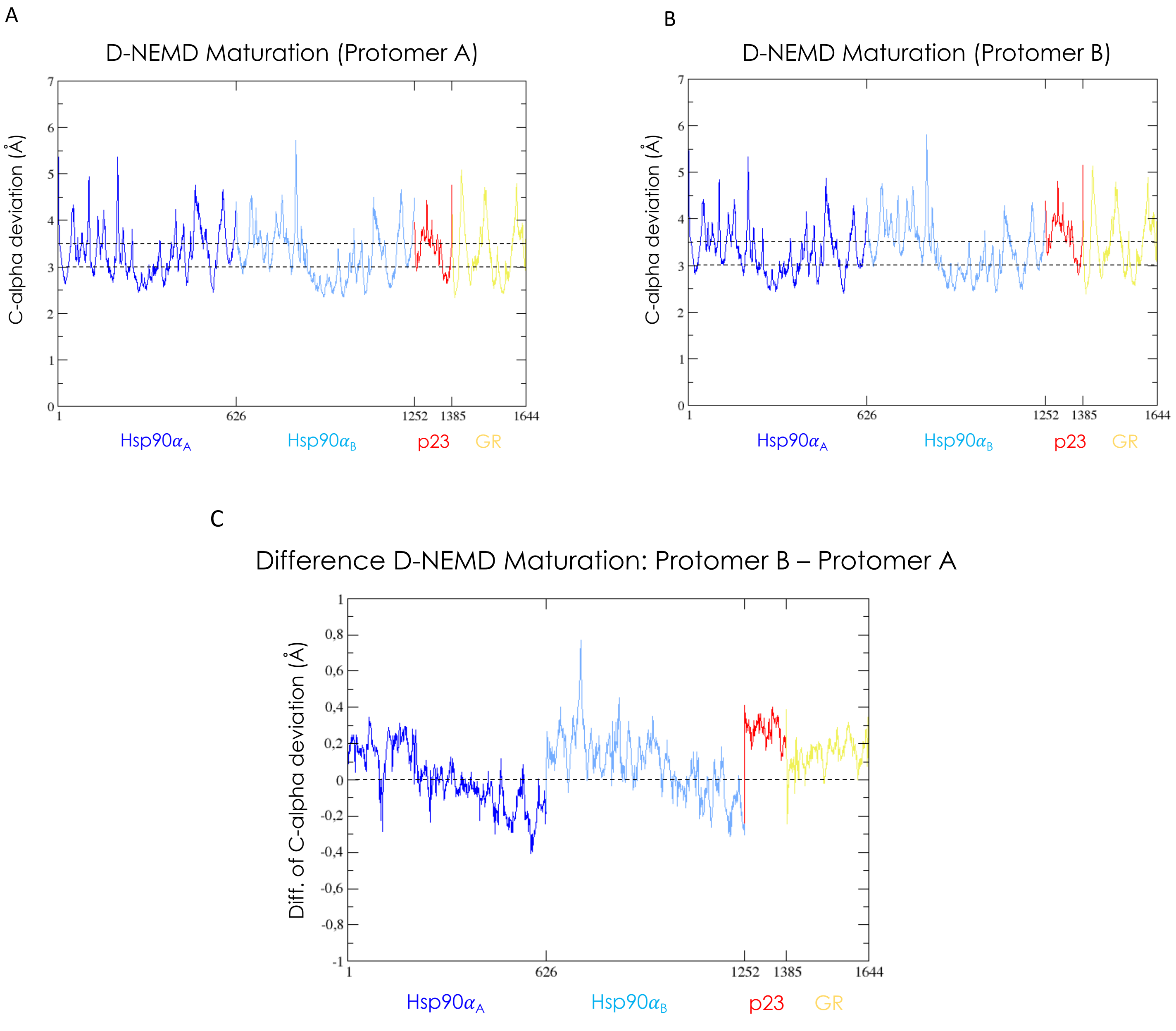

Figure S9. Average Cα deviation (Å) for each residues between the equilibrium Maturation-ATP simulations and the non-equilibrium ADP simulations. **(A)** Analysis for protomer A. **(B)** Analysis for protomer B. The average deviation is obtained from the 176 non-equilibrium simulations windows at 4 ns after ATP hydrolysis removal (see *Method* for details). On the x-axis are reported the single components of the multiprotein complex (the numeration corresponds to the one used in our simulations). **(C)** Difference calculated between Cα deviation of protomer B and of protomer A to highlight the asymmetric nucleotide signalling.

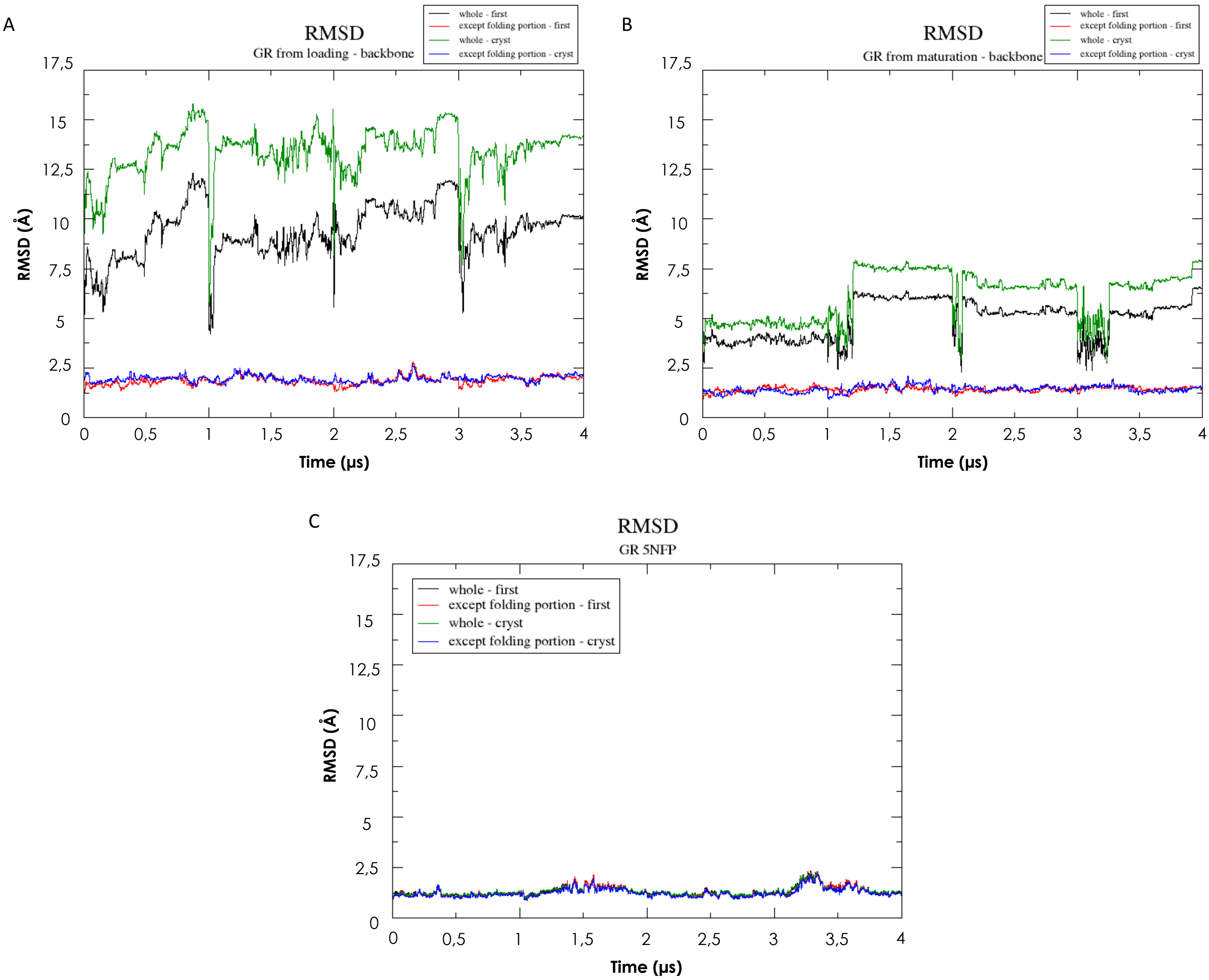

Figure S10. RMSD calculation (see *Methods* for details) of different GR systems: **(A)** GR from Loading complex **(B)** GR from Maturation complex and **(C)** GR active form (PDB: 5NFP).

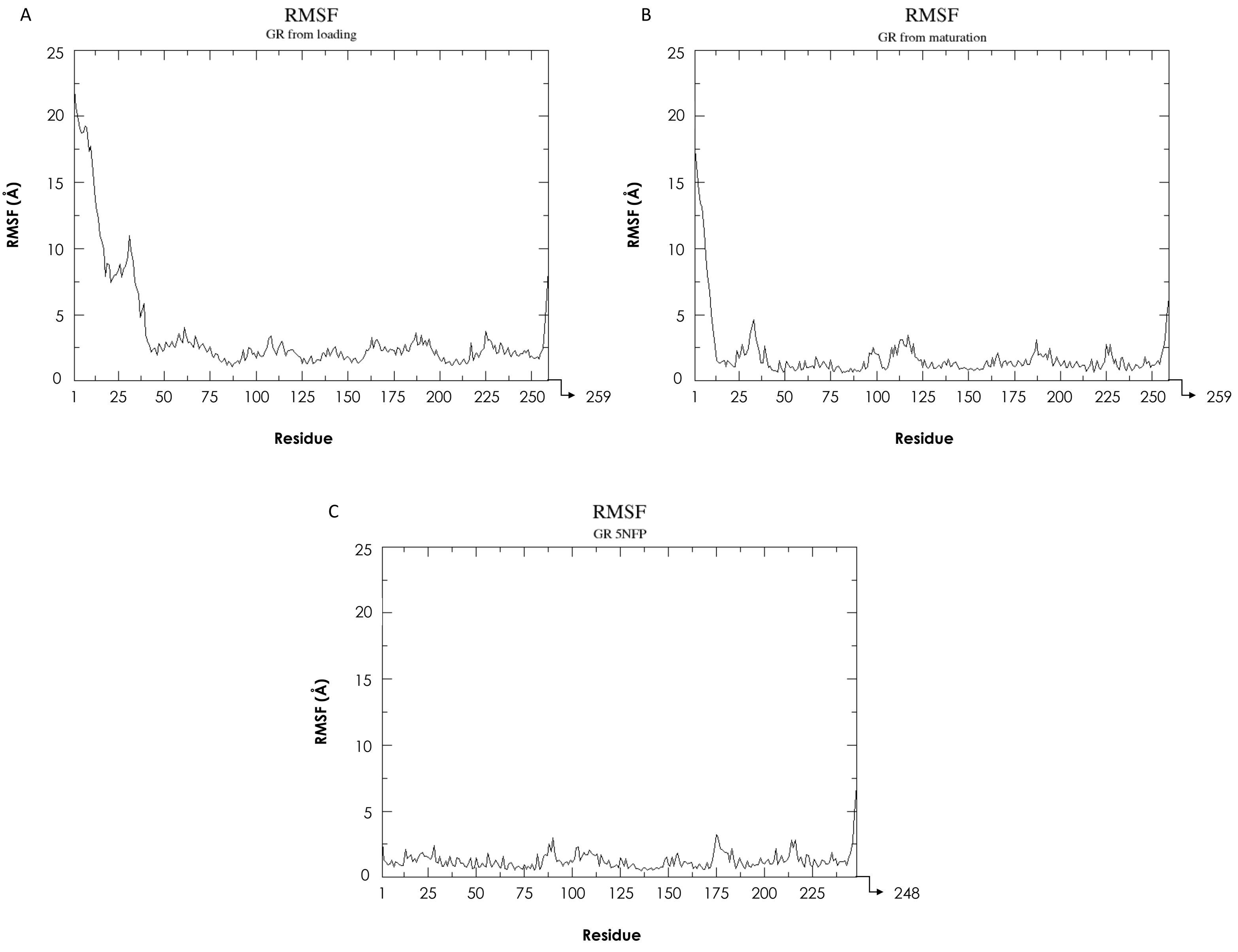

Figure S11. RMSF calculation (see *Methods* for details) of different GR systems: **(A)** GR from Loading complex **(B)** GR from Maturation complex and **(C)** GR active form (PDB: 5NFP).

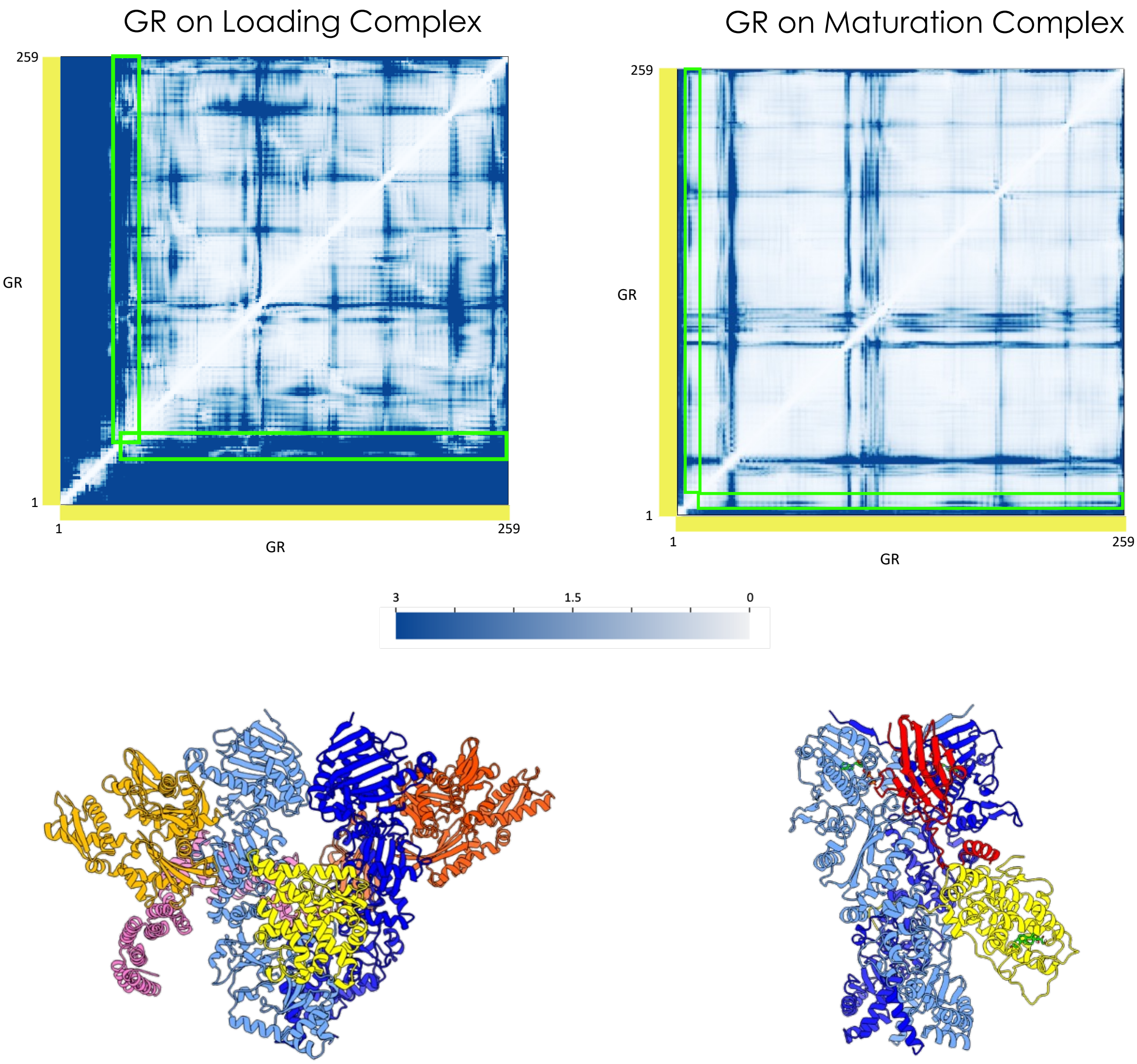

Figure S12. Distance Fluctuation pairwise matrices for the client GR simulations (extracted from Loading-ADP and Maturation simulation; see Table 1 for simulation labels). The axes report residue numbers. In the lower panel are reported the 3-D structures of GR in the two different systems. The green box in the matrices identifies the GR's tail regions that gain coordination in terms of DF values.

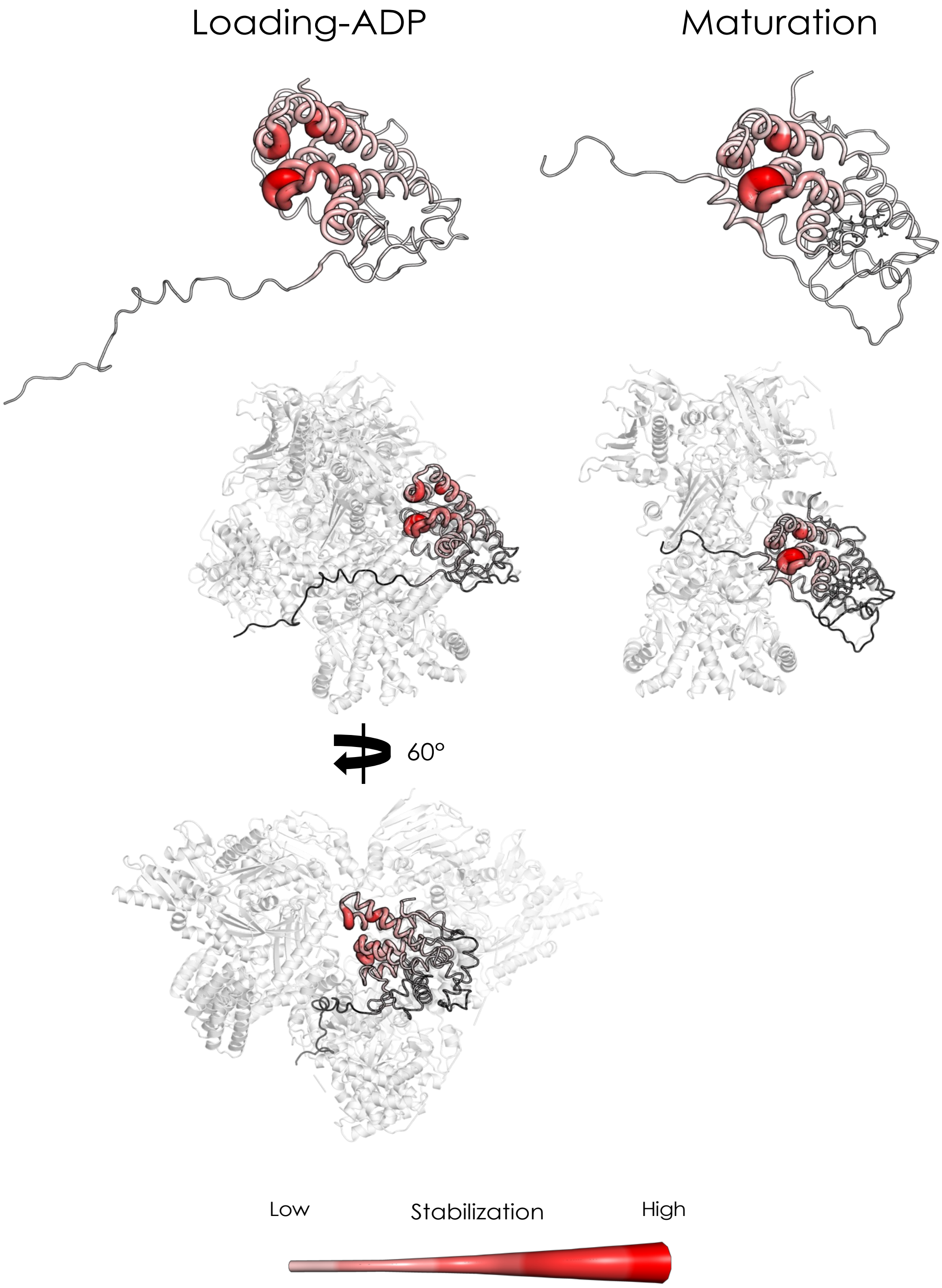

Figure S13. MLCE analysis on different GR state (left: Loading-ADP; right: Maturation). Each panel shows the regions that are energetically less prone to unfold, the folding core. Backbone thickness of each residue and colour intensity is proportional to the calculated energy coupling value (see *Methods*).

Table S1. List of residues that define the binding pockets identified for already known allosteric ligands for Hsp70 and Hsp90.

**HSP70 Ligands YK5 and MKT077** - L1262, T1265, Y1292, K1307, V1310, P1314, F1319, K1322, R1323, I1325, R1327, H1340, W1341, P1342, F1343, S1371, V1397, P1398, A1399, Y1400, E1426, P1427, I1448, D1450, G1453, T1455, F1456, S1459, T1473, G1475, D1476, T1477, G1481, E1482, K1508, R1512, R1515, T1516, E1519, K1522
**HSP70 Ligand YK5** - T1265, Y1266, A1281, N1282, Q1284, N1286, T1289, Y1292, I1302, D1304, A1305, K1307, N1308, A1311, L1312, K1377, R1509, R1512, R1513, R1515, T1516, A1517, R1520, R1523, A1531, S1532, L1533
**HSP90 Allosteric Ligand Pocket** - E935, H1005, E1006, D1007, S1008, R1011, Q1087, K1089, Q1677, I1678, Q1724, Q2433, P2436, Q2437, R2464, P2472, L2474, T2475
